# Deep learning and eye tracking: Convolutional neural networks provide converging evidence for experience-driven attention within visual search

**DOI:** 10.3758/s13428-026-03057-2

**Published:** 2026-06-04

**Authors:** Nicholas Crotty, Alenka Doyle, Kamilla Volkova, Nicole Massa, Noah C. Benson, Michael A. Grubb

**Affiliations:** 1 Trinity College, Hartford, CT USA; 2 eScience Institute, University of Washington, Seattle, WA USA

**Keywords:** Machine learning, Visual search, Eye movements, Experience-driven attention, Oculomotor data, Deep learning

## Abstract

Eye tracking during visual search generates spatiotemporally rich but complex data. Traditional analyses often utilize simplified metrics (saccade landings, dwell time, etc.) that necessarily exclude a substantial fraction of the variance in the raw eye data. Here, we asked if deep learning might aid scientists in objectively incorporating such discarded data into analyses. Convolutional neural networks (CNNs) are supervised machine learning tools that excel at classifying biological data. We built several CNNs that learn from raw eye-position time-course data to classify the location of relevant stimuli (e.g., search targets/distractors). We train each CNN on two-thirds of the data and cross-validate on the rest, comparing classification accuracy to chance via traditional frequentist testing and hierarchical Bayesian modeling. Using data from two of our previous visual search studies (Massa et al., Atten Percept Psychophys 86(4):1108–1119, 2024; Grubb & Li, Atten Percept Psychophys 80:822–828, 2018), CNNs successfully classified the location of distractors with a “history as a sought target,” finding evidence for reflexive, experience-driven overt attention within each oculomotor dataset. Successful prediction of distractor location generalized to a third dataset without additional training (Doyle et al., Atten Percept Psychophys 87:721–727, 2025) and outperformed a traditional saccade-landing metric. Feature visualization illustrated how the CNNs learn from eye-position samples near distractors early in trials and opposite distractors later in trials, suggestive of reflexive attentional allocations towards distractors followed by corrective shifts in gaze. We thus validate our CNN-based approach and highlight its utility in analyzing the spatiotemporally rich data gathered from eye tracking during visual search.

**Supplementary Information:**

The online version contains supplementary material available at 10.3758/s13428-026-03057-2.

## Introduction

High-resolution time-course data are important products of both psychophysical and neuroimaging experiments in vision. In particular, oculomotor time-course data from eye tracking is common in vision research and can be extremely rich in information. Because of this richness, analyses of oculomotor data are typically performed using heuristics and simplifying assumptions that drastically reduce the data complexity to ensure tractability. Heuristic algorithms are useful tools for probing dataset features, but their development is time-consuming, is typically based on a small subset of the dataset’s features, and requires arbitrary decisions by the researcher that could bias a particular aspect of the oculomotor information while ignoring others. Recent advances in machine learning (ML) have yielded an explosion of methods, such as convolutional neural networks (CNNs), that are well suited to modeling complex yet data-rich biological processes. CNNs are a form of neural network that transform an input using sequential layers of densely spaced computational filters (e.g., Benson et al. 2025; Kruper et al., 2024; LeCun et al., 1989; for a review, see Lindsay, 2021). Although CNNs fundamentally produce heuristic algorithms themselves, they are capable of doing so quickly, at scale, and in a manner that is informed by all samples in a dataset. Whereas human researchers often create heuristics by focusing on a small subset of the data (i.e., specific features), CNNs are trained on the entire dataset diffusely, reducing the probability that a CNN’s heuristic will perform well on only a subset of the data. Furthermore, CNNs can exploit far greater richness and complexity within a dataset because (as ML algorithms) they assess many randomly chosen heuristics against the whole dataset efficiently and without the need for human intervention. Here we apply CNNs to oculomotor time-course data to ask whether a CNN can learn to predict the location of relevant search objects (e.g., targets and/or distractors) from raw oculomotor data, evidencing the allocation of overt attention to such objects.

In a typical visual search task, participants are presented with a set of multiple stimuli, called the “search array.” Participants make a series of eye movements to visually locate a specific stimulus (the search target) within the array and then make a perceptual judgment (e.g.*, *whether the line inside the target circle is vertical or horizontal). Continuous eye tracking during visual search captures moment-by-moment changes in eye position, yielding an additional empirical data source beyond the speed and accuracy of the perceptual decision. Such eye tracking data are spatiotemporally rich but also complex. In the context of visual search, it is common practice to derive a simplified metric to reduce this complexity (Godwin et al., 2021; Hollingworth & Bahle, 2020). For example, many visual search studies calculate proportions of trials based on the landing location of the first recorded saccade, or quantify the dwell time of gaze in a specific area of the array (e.g., Anderson & Yantis, 2012; Becker et al., 2009; Gaspelin et al., 2016; for a review, see Godwin et al., 2021). However, such simplifications inadvertently exclude aspects of the oculomotor data that could prove informative about ongoing psychological processes. An alternative way to deal with the complexity of continuous eye tracking data would be to leverage advances in ML by training a CNN to learn from the entire time-course.

Because there are often multiple fixed locations where stimuli can appear on each trial in a visual search experiment, the problem we aim to solve is fundamentally a classification problem. Many kinds of CNNs can be trained to classify an input according to the most likely of two (binary classification) or more (multilevel classification) distinct categories (for a review, see Gu et al., 2018). In the context of visual search, a CNN can be given a time-course of eye position data as an input and be trained to predict the location of a search object (such as targets, distractors, or other relevant stimuli) as its output (i.e., to perform multilevel classification). As an example, suppose a CNN was trained to predict the location of a task-irrelevant distractor from raw eye traces. Such a CNN would produce a set of values for each trial, with each value corresponding to the probability that the distractor was presented at a given location. The largest value in this set reflects the CNN’s best estimate of where the distractor was on a given trial. If this estimate matches the actual location of the distractor on that trial, then the CNN's classification is considered correct; otherwise it is considered incorrect. During learning, the training regime updates the CNN's parameters to optimize its classification accuracy, usually via an optimization routine such as gradient descent. To ensure that the CNN’s trained parameters are generalizable after learning, the network is typically cross-validated on a subset of the data that was held out during training. Importantly, the CNN’s parameters are held constant when classifying this validation set. The proportion of correct classifications in the validation set can serve as an overall accuracy metric of CNN performance.

After applying CNNs to oculomotor data in the manner described above, we can determine what spatiotemporal features in the eye trace were most informative for classification. While the above performance metric describes whether a CNN could detect an oculomotor signal for attentional allocation towards distractors, it provides no information on what that signal actually is. To indirectly characterize this signal, classification accuracy can be compared to traditional oculomotor metrics, determining whether the information used for CNN predictions is entirely captured by pre-existing techniques. For a more direct comparison, feature visualization techniques such as SHapley Additive exPlanation (SHAP) analysis (H. Chen et al., 2023; Lundberg & Lee, 2017) quantify each input feature’s contribution to a model’s output prediction. A SHAP analysis in the current context can quantify the contribution of each eye position sample within every oculomotor trace to the network’s overall prediction. These “SHAP values” can illustrate the spatiotemporal patterns that the CNN learns from to classify distractors, which can in turn be compared to known oculomotor phenomena during search.

Here, we use CNNs to classify the locations of distractors that elicit the allocation of “experience-driven attention” within three pre-existing visual search datasets from our lab (Doyle et al., 2025; Grubb & Li, 2018; Massa et al., 2024). These experiments were divided into two phases, with a search target defined by its color in the first phase and by its shape in the second phase. In each phase, participants located the target among five uniquely colored distractors and reported the orientation of a line contained inside. In the second phase, one of the distractor elements was rendered in a color that matched the target in the first phase. These distractors were neither task-relevant nor physically salient, but nonetheless elicited reflexive shifts of overt attention due to a “history as a sought target” (Anderson et al., 2021; but see Doyle et al., 2025, showing that associations with instrumental information may be the real driver). If participants reflexively allocate overt attention to such distractors, evidence for these attentional shifts should be present within the raw eye trace, from which, we hypothesized, a CNN would be able to learn. We find that CNNs successfully predict the location of both search targets (as a preliminary “sanity check”) and task-irrelevant distractors in Massa et al. (2024) and Grubb and Li (2018), as confirmed through traditional frequentist testing and hierarchical Bayesian modeling. Moreover, a CNN trained on the eye data from Massa et al. (2024) is able to predict distractor location using an entirely different set of eye traces from Doyle et al. (2025), evidencing a generalizable oculomotor signature of attention from which the network learns. The networks learned from oculomotor information besides the landing of first saccades, indicated by comparisons between CNN accuracy and a traditional saccade-based metric. A SHAP analysis indicated that the CNNs learn from eye position values near distractors early in the trial (i.e.*,* a reflexive allocation of experience-driven attention) and opposite distractors later in the trial (i.e.*,* a corrective shift in gaze away from distractors). We thus confirm the validity of a CNN-based approach and highlight its utility in analyzing the spatiotemporally rich data that arise from eye tracking during visual search studies.

## Methods

### Apparatus

For all datasets (Doyle et al., 2025; Grubb & Li, 2018; Massa et al., 2024), the perceptual tasks were programmed in PsychoPy (Pierce & MacAskill, 2018; https://www.psychopy.org/) and run on a Mac Mini. Stimuli were displayed on a 27.0-inch LED-lit Dell gaming monitor (model S2716DG), with screen resolution of 2,560 × 1,440 pixels. Participants were seated in a darkened experimental testing room and indicated their response via a Logitech F310 gaming controller.

### Perceptual tasks

**Fig. 1:**
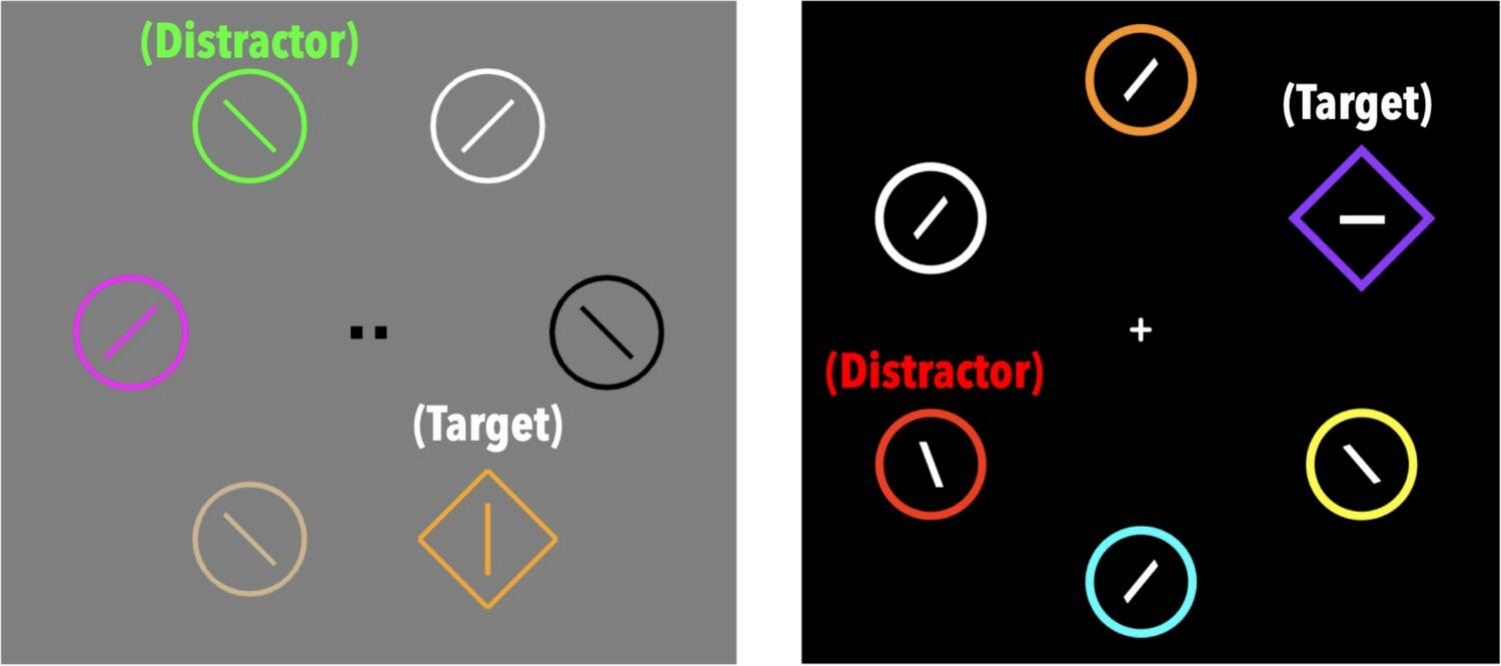
Example search arrays from Massa et al. (2024) and Grubb and Li (2018). These search arrays were displayed during the second phase of each study (Massa et al., 2024 [left], Grubb & Li, 2018 [right]). The search array displayed during the second phase of Doyle et al. (2025) was identical to that of Massa et al. (2024). Labels of search objects have been added for ease of understanding, and were not present during the actual experiments

We used data previously published by Massa et al. (2024) in which 79 participants performed a two-part visual search experiment. Specifically, we used the oculomotor dataset from the second phase (Fig. 1, left), where participants performed 480 trials each in which they reported the orientation of a line (two-alternative forced choice, vertical or horizontal) within a shape-defined target (a diamond among circles or a circle among diamonds) among five distractor shapes, each one rendered in a unique color. These stimuli were positioned in a circular arrangement around fixation, and were equidistant from the center. Within this array of six stimuli, two were positioned on the horizontal meridian, with the other four equally spaced between them. Participants had 1,200 ms to indicate their response. On half of all trials, one of the distractors was rendered in one of the colors previously indicative of the search target in the first phase (red, lime, yellow, or cyan, which appeared in equal proportions during the first phase). On the other half of second-phase trials, none of the distractors were rendered in a color used for the first-phase search target. When present, this distractor was equally likely to appear at any of the five locations in the search array not taken up by the search target. (*Note:* The first phase manipulated associations between target color and reward magnitude, as well as associations between target color and instrumental information. While we did find differences in the degree to which information associations modulated attentional capture, all distractors showed evidence of biasing eye movements. Thus, for the purposes of the current manuscript, we do not differentiate between distractor type.) For additional methodological detail, see the previous publication (Massa et al., 2024).

We also model the data reported by Grubb and Li (2018), in which 104 participants performed a two-part visual search experiment^1^. We used the oculomotor dataset arising from the second phase (Fig. 1, right), where participants performed 240 trials in which they reported the orientation of a line (two-alternative forced choice, vertical or horizontal) within a shape-defined target (a diamond among circles or a circle among diamonds) among five distractors, each one rendered in a unique color. These stimuli were positioned in a circular arrangement around fixation, and were equidistant from the center. Within this array of six stimuli, two were positioned on the vertical meridian, with the other four equally spaced between them. Participants had 1,200 ms to indicate their response. In half of the trials, one of the distractors was rendered in a color indicative of the search target in the first phase (red or green, which appeared in equal proportions during the first phase). In the other half, none of the distractors were rendered in a color used for the first-phase search target. When present, this distractor was equally likely to appear at any of the five locations in the search array not taken up by the search target. Grubb and Li (2018) consisted of a background study and a registered report, which had identical protocols save for a change in the viewing distance relative to the monitor^2^ and a feedback manipulation. For additional methodological detail, see the previous publication (Grubb & Li, 2018).

We also utilize oculomotor data from Doyle et al. (2025), in which 120 participants performed a modified version of the two-part visual search experiment from Massa et al. (2024). The associated experimental protocol was identical to that of Massa et al. (2024), save for the following: (1) As opposed to the trial-to-trial reward received for correct responses in the former study, participants in Doyle et al. (2025) solely received accuracy-based feedback, and were provided a fixed amount of compensation at the study’s end. (2) If any current observers had previously participated in Massa et al. (2024), their data were excluded prior to analysis, preventing any residual associations from impacting results. For additional methodological detail, see the previous publication (Doyle et al., 2025).

### Eye tracking

Eye movements were recorded in both studies using an EyeLink 1000 infrared-video eye tracker (SR Research, Ottawa, Ontario, Canada), with a nine-point calibration routine performed prior to starting each phase of each study. In the datasets from Massa et al. (2024) and Doyle et al. (2025), participants kept their chins in a chin rest located 70 cm from the monitor. For the background study within Grubb and Li (2018), participants were allowed to move their head freely while seated ~96 cm from the monitor, with the eye tracker set to “remote viewing mode.” For the registered report within Grubb and Li (2018), participants kept their chins in a chin rest located 60 cm from the monitor. We used the *eyelinkReader* package to import all resulting EDF files in R Studio (GitHub: alexander-pastukhov/eyelinkReader).

### Exclusion criteria

For each participant, we calculated the proportion of samples in which eye position was missing while the stimulus array was presented in each trial. We excluded participants with an average proportion of missing data greater than two standard deviations above the group average. Applying this exclusion criteria to the Massa et al. (2024) dataset excluded seven participants, leaving oculomotor data from 72 participants for the analysis of the second phase. Applying this exclusion criteria to the second phase of Grubb and Li (2018) dataset excluded data from seven participants, leaving oculomotor data from 97 participants to use in the current analyses. Applying this exclusion criteria to the second phase of Doyle et al. (2025) excluded data from 10 participants, leaving data from 110 participants to use with our approach.

### Data preprocessing

Across both datasets, *x*-position and *y*-position of participants’ gazes were sampled at a rate of 500 Hz, with each feature (*x*- and *y*-position of gaze in pixels) stored as a time-course vector. Eye position during the search array presentation was extracted for each trial and for each participant. Any missing eye samples (indicated by NAs within the unprocessed eye data) were replaced with the appropriate value of central fixation (1,279.5 pixels for *x*-position vectors, 719.5 pixels for *y*-position vectors). Given that the search array disappeared when a response was made, extra samples with the appropriate fixation coordinate were padded at the end of each trial’s time-course to equate vector length (600 samples for the 1,200-ms window of each study). If any time-course vectors were longer than the corresponding maximum (due to slight temporal misalignments between the sampling rate of the eye tracker and the signal indicating the response window’s end), the excess samples were removed. This preprocessing resulted in two complete time-course vectors of maximum length, one containing the *x*-position and one containing the *y*-position, for each trial of every participant. These vectors were then concatenated into a two-dimensional array of eye position data for each feature, creating an array with only *x*-position data and an array with only *y*-position data. Within these arrays, the rows represented trials, and the columns represented samples. Upon being uploaded into the analysis script, the two-dimensional arrays of trial-level eye position samples were then transformed into a three-dimensional array, with the array’s rows, columns, and slices reflecting the trials, features, and samples of the entire preprocessed oculomotor dataset, respectively.

For neural networks forming predictions about target location, labels denoting the actual location of the search target within the array were also created for each trial. These labels consisted of a single number representing the target location through numerical encoding on a range of 0–5 (representing all six potential target locations). During the formation of the feature arrays, the labels were concatenated into a one-dimensional array (*x*-axis, trials) in the same order as their corresponding eye position data. These labels were eventually compared to the outputs of the applied neural networks to assess if their formed predictions were correct (see “CNN Architecture” for more information). For neural networks predicting distractor location, this procedure was identical, but with the location of the distractor saved rather than the target.

### Training/validation split

For all CNNs predicting target location, preprocessed trial-level eye position data were divided into a two-thirds/one-third training/validation split (Joseph, 2022). Every third trial was extracted from each participant’s dataset to form the validation set. The remaining trials comprised the training set. For neural networks predicting distractor location, distractor-absent trials were first excluded, then the two-thirds/one-third training/validation split was performed on the resulting subset of distractor-present trials. In the demonstration of model generalizability with the CNN trained on the Massa et al. (2024) data (see “Results” for more information), the entire eye position dataset from Doyle et al. (2025) was provided for validation, without the need for a training/validation split.

### Mini-batching

Trials within each training dataset and their corresponding labels were divided into groups, or “mini-batches” of 64 prior to being received by a neural network. If a dataset was not exactly divisible by 64, the last mini-batch contained the remaining excess trials. As opposed to only updating its parameters after experiencing the entire dataset (which would lead to only one parameter update per epoch), this mini-batching technique allowed the applied neural network to update its parameters after each batch (for a total of $\left\lceil n/64\right\rceil$ parameter updates, rounded up as shown by the ceiling delimiters, where $n$ is the number of trials in the entire dataset), increasing its pace of learning.

### CNN architecture

**Fig. 2:**
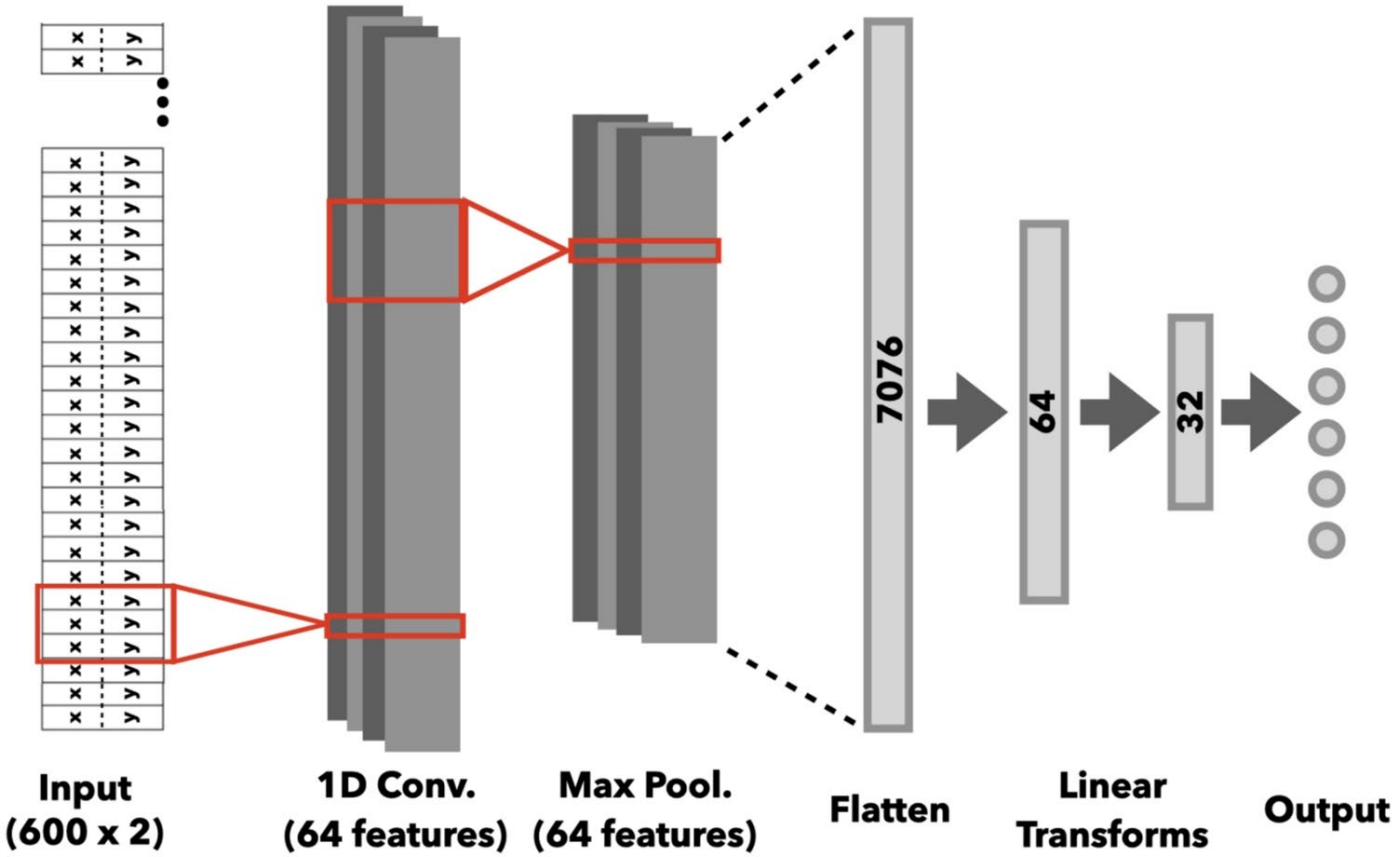
CNN architecture. Schematic illustrating the architecture of all CNNs used in the reported analyses. Red boxes before triangles symbolize kernel size; red boxes after triangles symbolize size of kernel output. The numbers displayed on the linear layers and the dots in the output layer reflect the number of neurons in the corresponding layer. The trial-level input to the networks consisted of a time-course vector containing *x*- and *y*-position of gaze throughout the search array presentation on the given trial. For figure readability purposes, dimensions of individual layers are not drawn to scale

All neural network analyses were coded and performed using PyTorch (Ansel et al., 2024; https://pytorch.org/) within a Google Colab Notebook. We used a one-dimensional CNN consisting of one input layer, three neuron-containing hidden layers, and one output layer (Fig. 2). Within a mini-batch, time series trials representing eye position were processed one trial at a time by an input layer that performed a one-dimensional convolution between the trial’s values and the layer’s convolution kernel, whose weights are learned through training (Gu et al., 2018; Krizhevsky et al., 2012; Lindsay, 2021). This convolution kernel iteratively progressed through the input along its time-course. With each progression, the dot product of the kernel’s learned weights with a specific range of samples (denoted by the kernel size) was calculated. The kernel was then shifted by a certain number of samples (denoted by the stride) and another dot product was calculated, with this process repeated throughout the length of the input. Full implementation details of the CNNs used in this paper are included in the source code. In brief, the convolution layer used by the CNN had a kernel size of 3, and progressed through the input with a stride of 1. This convolution produced a two-dimensional output with 64 features. The output of the convolution layer was then passed through a rectified linear unit (ReLU) activation function, where ReLU(*x*) = *x* if *x* > 0, otherwise 0 (Krizhevsky et al., 2012). This output was in turn passed through a regularization method known as dropout, which reduces the potential for individual neurons within the CNN to over-rely on the values of other individual neurons by zeroing each value in a layer’s input with probability *p* (Bisong, 2019). We set the dropout rate (*p*) to 0.25, meaning that there is a 25% chance for each value within the input to be set to zero. After this regularization, the resulting output is passed through a maximum pooling function (Lindsay, 2021) with a size and stride of 5. Max pooling functions have no effect on the calculation of a CNN for a single input, but they increase a CNN’s training efficiency by focusing the search algorithm on the maximum values within regions of the input. This pooling produced another two-dimensional output with 64 features.

The resulting two-dimensional output was flattened into a one-dimensional sequence of 7,616 values and passed through multiple fully connected linear layers with no activation function, referred to in this case as linear transforms (Gu et al., 2018; Lindsay, 2021). There were three total linear transforms, with one containing 7,616 neurons, one containing 64 neurons, one containing 32 neurons, and the output transform containing six neurons, which produced six values (i.e., logits). These six output values were then directly compared to the target (or distractor) location label corresponding to the current trial. If the position of the largest logit within the output matched the number within the label, the CNN successfully predicted the location of the target from that trial’s eye position data. If the position of this largest logit did not match the number within the label, the CNN incorrectly predicted that trial’s target location.

### Loss function

After passing through each mini-batch of the training set, all neural networks update their learned weights based on their accuracy in predicting target (or distractor) location, in a process known as backpropagation (Krizhevsky et al., 2012; Rumelhart et al., 1986). The overall accuracy of a prediction is assessed using a loss function, which must decrease as the accuracy increases, similar to a negative likelihood function (Gu et al., 2018). We used categorical cross-entropy (Kullback & Leibler, 1951; Mao et al., 2023), a function commonly used with multilevel classification, as the loss function for all neural networks. When updating their parameters, the neural networks calculate the gradient of this loss function with respect to their current parameter values and scale this value using a coefficient known as the learning rate. This scaled value is then subtracted from the current parameter values, producing the updated weights used for the next iteration of learning.

### Early stopping

For all of our CNN-based analyses in which the networks were trained on data (i.e., save for the demonstration of model generalizability), we implemented an early stopping procedure. This procedure is designed to limit the possibility of a network overfitting to the training data by stopping the training/validation process once the observed loss on the validation set stops decreasing (Prechelt, 1998). We utilized an existing version of early stopping protocol designed for PyTorch implementation (Sunde, 2024). After each training/validation cycle, the CNN’s parameter weights would be saved if the validation loss reached a new minimum. If the validation loss did not reach a new minimum, then a counter that tracked the criteria for early stopping would be incremented by 1. Our early stopping protocol had a patience of 10, meaning that the training/validation cycle would cease after counting 10 consecutive epochs with no decrease in minimum validation loss. Once this criteria was met, the CNN with parameter weights that produced this minimum loss would be restored, as is common practice with early stopping (Bisong, 2019; Rice et al., 2020). We use the resulting classification accuracy from this iteration of the CNN in our statistical analyses.

### Exponential decay in learning rate

If a neural network progresses through training/validation cycles with a constant learning rate, the gradients calculated during backpropagation (see “Methods—Loss function” for more information) can become so large that they approach infinity, impairing the precision with which the network can converge on a solution (Hochreiter, 1991). To address such “exploding gradients,” we implemented an exponential decay in the learning rate (e.g., Benson et al., 2025). This approach decreases the magnitude of the learning rate at the end of every training/validation cycle by multiplying the learning rate with term $\gamma$, where $\gamma <1$. We utilized a $\gamma$ of 0.9, meaning that the learning rate would decrease to 90% of its previous magnitude after every cycle.

### Hyperparameters and AI ethics

Every CNN was provided trials via mini-batch, with a batch size of 64. The Adam optimizer (Kingma & Ba, 2015) was used for fitting CNNs to data. Since each network performed multilevel classification, categorical cross-entropy (Kullback & Leibler, 1951; Mao et al., 2023) was used as the loss function. The learning rate for all CNNs was initialized as 10^−3^, and decreased by a factor ($\gamma$) of 0.9 every cycle. We used an early stopping protocol with a patience of 10.

**Fig. 3:**
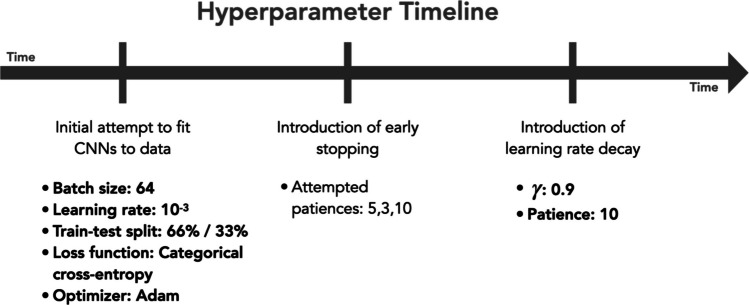
Hyperparameter timeline**.** Conceptual diagram illustrating hyperparameter choices across timeline of developing our CNN-based approach. Bold text denotes finalized hyperparameters that received no further tuning or optimization through the course of the study. See “Methods—Hyperparameters and AI ethics” for details

To promote open science practices within the context of machine learning, we report the order, timing, and process by which the above hyperparameters were set (Fig. 3). The loss function (categorical cross-entropy), batch size (64), and learning rate (10^**−**3^) were selected to match those used in the “Learn the Basics” tutorial from the PyTorch documentation (Ansel et al., 2024). The Adam optimizer was chosen based on a separate tutorial in the documentation devoted to different types of optimizers. These hyperparameters were set before fitting any CNNs to data, and were left unchanged throughout the course of the study.

Our early stopping protocol and exponential learning rate decay were incorporated at later time points in the project, and thus informed by preliminary attempts to fit CNNs to data. In the initial implementation of our approach, we observed high variability in CNN accuracy and loss between training/validation cycles, which we initially attributed to the presence of local minima during optimization. We then incorporated early stopping and arbitrarily chose several patience values of varying sizes to characterize this variability (see Fig. 3). A longer patience of 10 revealed a sudden and sustained divergence during fitting within later training/validation cycles. Continued review of the literature and communication between authors concluded that this high variability and eventual divergence was not due to local minima but was instead driven by “exploding gradients” (see “Methods—Exponential decay in learning rate”). To prevent the rapid increases in calculated gradients that produce said divergence, we incorporated an exponential decay of the learning rate into our approach, utilized in one author’s previous work (Benson et al., 2025). We arbitrarily chose a $\gamma$ of 0.9 for this analysis, with no optimization performed over this hyperparameter. Once this change was implemented to our approach, all hyperparameters were finalized and remained unchanged throughout the course of the study.

### Statistical analyses

*Paired t-tests*. For each neural network, the participant-level classification accuracies were compared to a vector containing chance performance (16.67%, random guessing between six potential target locations).

*Confidence intervals.* 95% confidence intervals for each CNN’s classification accuracy were computed using 10,000 bootstraps of the participant-level CNN accuracies.

*Hierarchical Bayesian modeling*. For each CNN, we applied a hierarchical Bayesian model to the binary classification outcomes (i.e., successful or unsuccessful) for the validation set (Eq. 1). We modeled the number of correct classifications, *C*, for each individual, *i*, as the result of *N* draws from a binomial distribution with probability *p* (Eq. 1, line 1). We define *p* for each individual as a random draw from a beta distribution with shape parameters *α* and *β* (Eq. 1, line 2). We assign weakly informative priors to each of these shape parameters (Eq. 1, lines 3 and 4) to center the prior of the beta distribution closer to chance. We utilized Hamiltonian Monte Carlo (Duane et al., 1987), running four chains of 1,000 iterations each for all models, which was performed using the rethinking package in R (McElreath, 2020). All models will be made publicly available, along with a corresponding spreadsheet providing detailed information about all posterior distributions, including mean, standard deviation, 95% credible interval, number of effective samples, and $\widehat{R}$ (a metric used with Monte Carlo algorithms for determining convergence).

1 $$\begin{array}{l}\begin{array}{l}\begin{array}{cc}1& {C}_{i} \sim \end{array} \mathrm{binomial}\left({\mathrm{N}}_{i},{p}_{i}\right)\\ \begin{array}{cc}2& {p}_{i}\end{array}\sim \mathrm{beta}\left(\alpha ,\beta \right)\\ \begin{array}{cc}3& \alpha \end{array}\sim \mathrm{exponential} (0.1)\end{array}\\ \begin{array}{cc}4& \beta \end{array}\sim \mathrm{exponential} (0.05)\end{array}$$

### SHAP analysis

We used SHapley Additive exPlanation (SHAP) analysis to visualize how the CNNs classifying target location learned from input data and form their predictions. Arising from concepts in game theory, SHAP values are an additive metric that quantifies each input feature’s contribution to a model’s output prediction, relative to an “explainer model” applied to a subset of the data (Lundberg & Lee, 2017). A SHAP analysis was performed on the validation set from each CNN-based analysis, both those predicting target location and those predicting distractor location. We used the "Deep SHAP” approach specific to networks with hidden layers (H. Chen et al., 2023), in which the SHAP values are approximated through the DeepLIFT algorithm (Shrikumar et al., 2017). Through the SHAP library (GitHub: shap/shap), we applied the explainer to the first 100 validation trials, then calculated SHAP values for each eye position sample within every trial-level time-course of the validation set. For each of these samples, 12 values were calculated: six representing the contribution of the *x*-position to predicting each of the potential object locations, and six representing the contribution of the *y*-position to predicting each potential location. We then took the mean absolute value across classes and across features to produce a single “global feature importance” metric for each sample, referred to as a “SHAP value” in the subsequent analyses.

### Gaussian-weighted kernel density estimate

To precisely visualize the spatial regions that were most informative for target classification, we performed a Gaussian-weighted kernel density estimate, or KDE, to the trial-level maximum SHAP values, after rotating each trial’s eye trace to align target locations (see Supplementary Methods for more information). In short, KDE attempts to characterize the underlying density function for a given dataset by “smoothing” each data point into a known symmetric function (in this case a Gaussian), then summing the resulting set of distributions (Y.-C. Chen, 2017; Silverman, 1986). Kernel bandwidth (i.e*.*, the relative spread of each symmetric function) was calculated using Silverman’s rule of thumb (Silverman, 1986), which incorporates both the number of coordinates and their standard deviation (averaged across *x*- and *y*-dimensions). A resolution of 10 pixels was used when plotting the resulting KDE as a density heatmap, with the density values normalized to allow comparison between heatmaps.

## Results

### Predicting the location of search targets: a preliminary “sanity check”

#### Frequentist interpretation of CNN accuracy

**Fig. 4:**
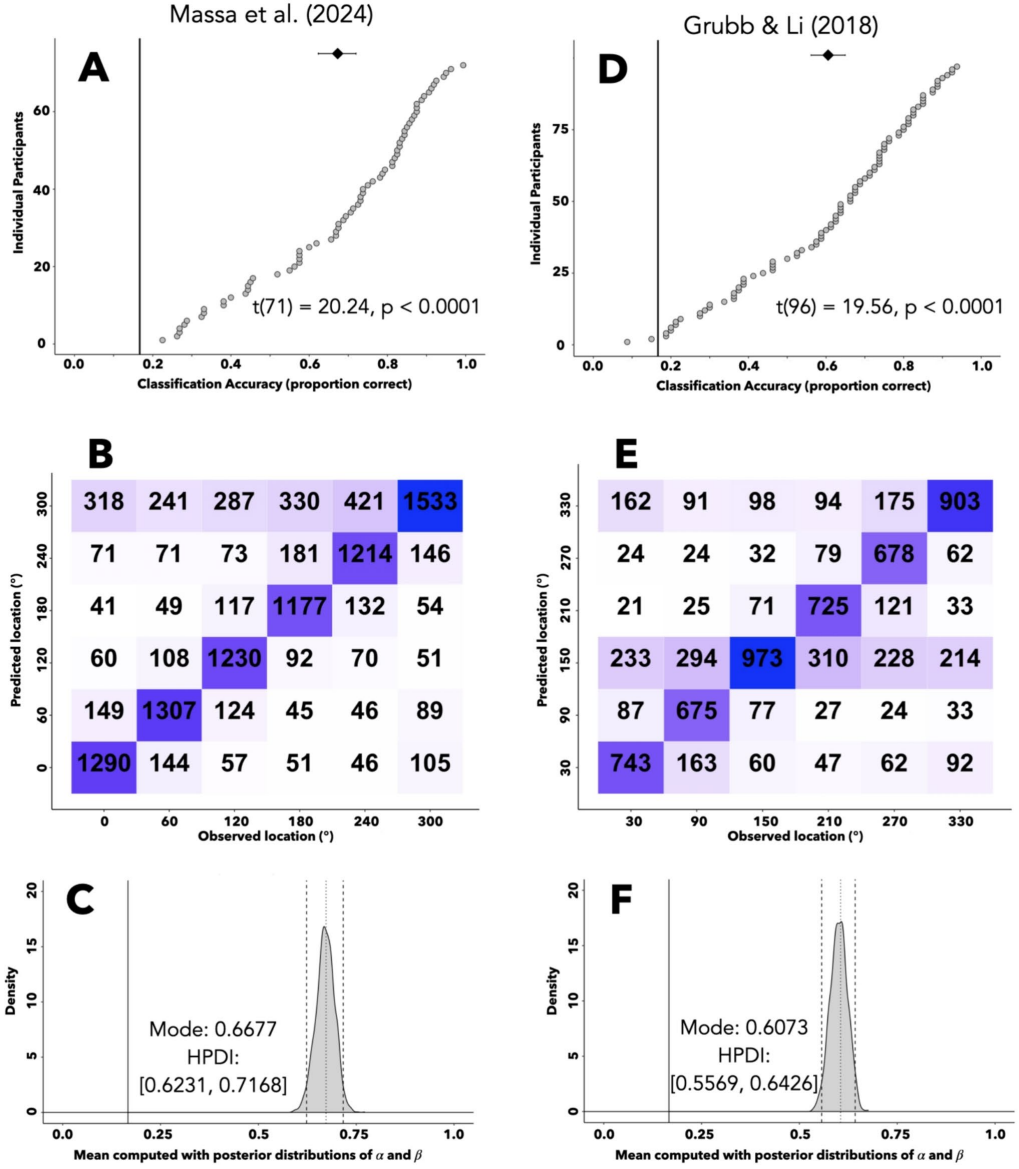
Predicting target location. **A**, **C** Classification accuracy of a CNN predicting the location of a search target using eye position data from the Massa et al. (2024) or Grubb and Li (2018) dataset (**A** and **C**, respectively). Circles depict participant-level accuracies sorted in ascending order. Diamond depicts mean classification accuracy, with a 95% confidence interval bootstrapped at the participant level. Solid line denotes chance accuracy. **B**, **D** Posterior distribution describing the mean of the beta distribution used to generate the participant-level probabilities of a CNN correctly predicting target location in the Massa et al. (2024) or Grubb and Li (2018) dataset (**B** and **D**, respectively). This mean was computed using the posterior distributions of shape parameters *α* and *β*, derived from a hierarchical Bayesian model

If there is any hope of using raw eye traces to correctly classify the location of task-irrelevant distractors, we should be able to classify the location of the task-*relevant* target, which is expected to be overtly attended on each trial. Indeed, convolutional neural networks (CNNs) learned to successfully classify the location of visual search targets using only the information contained in raw eye traces. We fit identical CNNs to the data from the second phases of Massa et al. (2024) via a series of training/validation cycles. Each cycle consisted of a round of training through the training dataset, after which the CNN’s parameter weights were frozen, and the network was tested on the validation trials. The Massa et al. (2024) dataset consisted of 23,040 training trials and 11,520 validation trials. Early stopping criteria was met after 47 training/validation cycles and the parameter weights from the 37th iteration (i.e., the cycle with the smallest validation loss) were restored. The CNN then correctly classified target location on 7,751 validation trials for an overall classification accuracy of 67.28%, well above the expected value produced by randomly guessing between six potential target locations (1/6 = 16.67%). The mean participant-level accuracies (Fig. 4A) were significantly greater than chance, *t*(71) = 20.24, *p* < 0.0001, and the mean across participants^3^ (67.28%) has a participant-level, bootstrapped 95% confidence interval that is far from chance [62.32–72.06%]. The network had relatively similar classification accuracies across the six different potential target locations, as visualized by the confusion matrix in Fig. 4B.

Another CNN learned to successfully predict the location of search targets using eye position data from Grubb and Li (2018). This network was trained on the 15,520 training trials of the Grubb and Li (2018) dataset and tested on the 7,760 validation trials. Early stopping criteria was met after 55 rounds of training and the weights from the 45th round were restored. The network then correctly predicted target location 4,697 times (60.53% accuracy). The mean accuracy across participants (60.53%, Fig. 4D) has a bootstrapped participant-level confidence interval well above chance [56.19–64.81%] and a one-sample *t*-test indicates that the participant-level mean accuracies are significantly greater than chance, *t*(96) = 19.56, *p* < 0.0001. Network classification accuracy was relatively similar across potential target locations (Fig. 4E).

#### Bayesian interpretation of CNN accuracy

Some readers may prefer a Bayesian approach to inference. To assess whether a given CNN classified target location better than chance, we fit a hierarchical Bayesian model to the binary trial-level outcomes of each CNN's validation dataset (i.e., successful or unsuccessful classification). Our model assumes the following: (1) Each participant’s eye position data contain information from which the CNN can correctly classify the target location with a probability that is unique to each individual, *p*_*i*_ (see Methods, Model 1, Line 1). (2) The distribution of *p*_*i*_ values are generated by a beta distribution with shape parameters *α* and *β* (Model 1, Line 2). We used weakly informative prior distributions and Hamiltonian Monte Carlo to estimate the posterior distributions of *α* and *β*, from which the mean of the beta distribution defined by these parameters could be computed ($\mathrm{mean} =\frac{\alpha }{\alpha +\beta }$). Figure 4C plots the posterior distribution for the CNN fit to the Massa et al. (2024) dataset, showing that we can be 95% confident that the beta distribution from which our *p*_*i*_ values originated has a mean that falls between 0.62 and 0.72 (dashed lines). The 95% high-probability density interval (HPDI, dashed lines) is far from chance (solid line), as indicated by the dashed lines not including the solid line. Additionally, the empirically observed proportion correct value (dotted line) falls neatly in the center of this HPDI, evidencing a good fit between the data and the model. The posterior distribution for the CNN fit to the Grubb and Li (2018) dataset is shown in Fig. 4F. Here, the empirically measured proportion correct value (dotted line) was positioned near the center of the 95% HPDI (dashed lines), indicating a good fit, and the HPDI [0.56, 0.64] did not include chance (solid line), indicating a successful classification of target location by the CNN.

### Interim discussion I

While the above results provide an important “sanity check” for our CNN-based analysis approach, these conclusions could be derived through other simpler forms of analysis. For example, a fixed algorithm that calculates the average distance from participants’ gaze to each potential target location and returns the minimum can also identify target location using time-course eye position data (see Supplementary Results for an example). However, if one were to design a similar algorithm to predict *distractor* location using time-course data, the information or heuristic used to return the algorithm’s “guess” is relatively unintuitive. A neural network, on the other hand, can independently learn to prioritize evidence within the eye data that is relevant to a prediction, in this case classifying the location of a distractor.

In the next section, we highlight the key contribution of this paper: the ability of our developed CNN-based approach to predict the location of task-irrelevant distractors. In the second phases of both Massa et al. (2024) and Grubb and Li (2018), a task-irrelevant distractor was presented on half of all trials. If these distractors engender reflexive shifts in overt attention due to their “history as a sought target” (Anderson et al., 2021), evidence for such shifts should be present in the oculomotor data. Furthermore, if CNNs truly learn from an oculomotor signature of overt attention instead of participant-specific idiosyncrasies, a CNN trained on one dataset should be able to classify distractor location in another dataset without additional learning. We apply our CNN-based analysis to the distractor-present trials from each study, reporting frequentist and Bayesian interpretations of the networks’ accuracy in classifying distractor location. We also use the learned parameter weights from the CNN trained with Massa et al. (2024) data to predict distractor location in Doyle et al. (2025), as an indicator of whether the network learned from a generalizable oculomotor signal of reflexive overt attention. We then compare CNN accuracy to first saccade landings, an established metric for quantifying oculomotor effects of distractors (Doyle et al., 2025; Massa et al., 2024; Sauter et al., 2021; Wang et al., 2019).

### Can the CNNs successfully predict the location of experience-driven distractors?

#### Predicting the location of task-irrelevant distractors

**Fig. 5:**
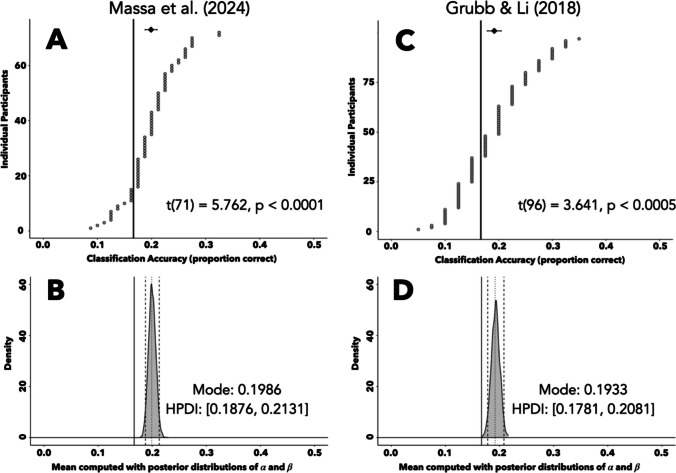
Predicting distractor location. **A**, **C** Classification accuracy of a CNN using eye position data from the Massa et al. (2024) or Grubb and Li (2018) dataset (**A** and **C**, respectively) to predict the location of a task-irrelevant distractor. Circles depict participant-level accuracies sorted in ascending order. Diamond depicts mean classification accuracy, with a 95% confidence interval bootstrapped at the participant level. Solid line denotes chance accuracy. The *x*-axes are truncated at 0.5 for figure readability purposes. **B**, **D** Posterior distribution describing the mean of the beta distribution used to generate the participant-level probabilities of a CNN correctly predicting distractor location in the Massa et al. (2024) or Grubb and Li (2018) dataset (**B** and **D**, respectively). This mean was computed using the posterior distributions of shape parameters *α* and *β*, derived from a hierarchical Bayesian model. The *x*-axes are truncated at 0.5 for figure readability purposes

A CNN learned to successfully classify the location of a task-irrelevant distractor using only the information contained in a time-course of raw eye position data. We fit a new CNN to the subset of data from Massa et al. (2024) containing distractor-present trials and compared the output logits to the location of the distractor (rather than that of the target, as done in the previous section). The CNN met early stopping criteria after 64 cycles through the dataset, after which the weights from the 54th cycle were restored. The network then correctly predicted distractor location on 1,147 of the 5,760 validation trials for an accuracy of 19.91%. At the participant level (Fig. 5A), the CNN performed above chance for data from 57/72 participants. The mean accuracy across participants (19.91%) had a participant-level bootstrapped confidence interval greater than chance [18.82–21.01%], and a one-sample *t*-test confirmed that the participant-level mean accuracies were significantly above chance, *t*(71) = 5.762, *p* < 0.0001.

The success of the CNN-based approach in predicting distractor location was also demonstrated via hierarchical Bayesian modeling. Mirroring the previous Bayesian modeling procedures, we applied a hierarchical model to the binary classification outcomes, with the fit shape parameters used to compute the mean of the beta distribution that gives rise to the *p*_*i*_ values. This distribution of the mean is depicted in Fig. 5B, and has a 95% HPDI (dashed lines) ranging from 0.19 to 0.21. The empirically measured proportion correct value (dotted line) falls near the center of this HPDI, indicative of a good fit by the model. Since this HPDI does not include chance (solid line), we can conclude that the CNN demonstrated above-chance accuracy in predicting the location of the task-irrelevant distractor.

Another CNN learned to successfully predict the location of a task-irrelevant distractor using eye position data from Grubb and Li (2018). We again fit a new CNN to the subset of distractor-present trials from this dataset and compared the output logits to the distractor location. The CNN met early stopping criteria after 38 cycles through the dataset, after which the parameter weights from the 28th epoch were restored. The network then correctly predicted distractor location on 744 of the 3,880 validation trials for an accuracy of 19.18%. At the participant level (Fig. 5C), the CNN performed above chance for 60/97 participants. The across-participant mean classification accuracy (19.18%) had a confidence interval (bootstrapped at the participant level) that was above chance [17.84–20.54%], and the participant-level mean accuracies were significantly greater than chance, one-sample *t*-test, *t*(96) = 3.641, *p* < 0.0005. We again modeled the binary classification outcomes with our hierarchical Bayesian approach (Fig. 5D). The empirically measured proportion correct value (dotted line) fell near the center of the 95% HPDI (dashed lines), signaling a good model fit, and the HPDI [0.18, 0.21] does not include chance (solid line), indicating that the CNN demonstrated above-chance performance in predicting distractor location.

#### Network generalizability in predicting distractor location

The strongest way to provide evidence for distractor-induced shifts of overt attention using a CNN-based approach would be to demonstrate the generalizability of the network’s learned parameters. In short, can we train a CNN using data from one experiment to classify distractor location in an entirely different experiment? If so, we can be certain that the CNN is not learning from idiosyncratic oculomotor patterns specific to the participants who generated the training dataset. Below, we show evidence of such generalizability with the parameter weights from the CNN trained to predict distractor location from Massa et al. (2024). Specifically, we use a CNN with these parameter weights to classify distractor location in data from a recently published follow-up study (Doyle et al., 2025), without the need for additional training on this new dataset.

**Fig. 6:**
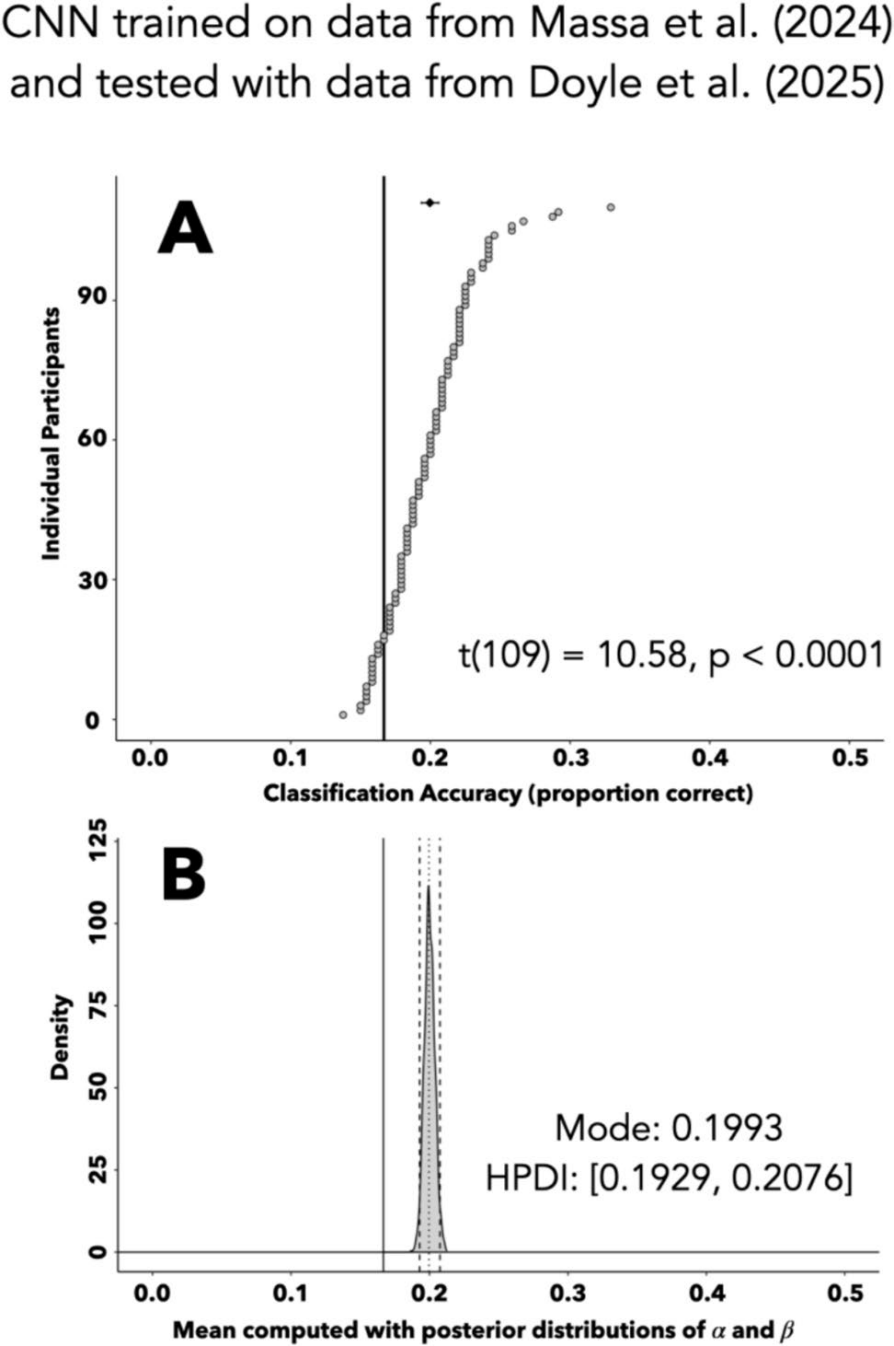
Predicting distractor location in an entirely new dataset. **A** Classification accuracy of a CNN using learned parameter weights from the Massa et al. (2024) dataset to predict the location of a task-irrelevant distractor in Doyle et al. (2025). Circles depict participant-level accuracies sorted in ascending order. Diamond depicts mean classification accuracy, with a 95% confidence interval bootstrapped at the participant level. Solid line denotes chance accuracy. The *x*-axis is truncated at 0.5 for figure readability purposes. **B** Posterior distribution describing the mean of the beta distribution used to generate the participant-level probabilities of a CNN trained on Massa et al. (2024) data and predicting distractor location in the Doyle et al. (2025) dataset. This mean was computed using the posterior distributions of shape parameters *α* and *β*, derived from a hierarchical Bayesian model. The *x*-axis is truncated at 0.5 for figure readability purposes

The CNN trained on the Massa et al. (2024) dataset classified distractor location on 5,270 of the 26,400 total trials from Doyle et al. (2025) with an accuracy of 19.96%. At the participant level (Fig. 6A), the network performed above chance for 92/110 participants. The across-participant mean accuracy (19.96%) had a participant-level confidence interval that was above chance [19.37–20.58%], and the participant-level mean accuracies were significantly greater than chance, *t*(109) = 10.58, *p* < 0.0001. We again applied our hierarchical Bayesian model to the binary trial-level classification outcomes (Fig. 6B). The model fit the data well, as indicated by the empirically measured proportion correct value (dotted line) falling near the center of the 95% HPDI (dashed lines), and the HPDI [0.19, 0.21] does not include chance (solid line). Thus, a CNN that was trained on eye data from Massa et al. (2024) successfully predicted distractor location using previously unseen raw eye position data from another study (Doyle et al., 2025), without the need for any additional learning.

#### Comparison of distractor-location CNN accuracy to traditional oculomotor metrics

While the previous results provide a compelling case for the CNN’s ability to detect and learn from overt attentional shifts towards task-irrelevant distractors during a search, they do not explain whether the CNNs are learning from information not easily captured by traditional oculomotor metrics. To address this open question, we compared CNN performance to the landing of the first saccade on each trial, which has been traditionally used to quantify distractor-induced shifts of overt attention (e.g., Sauter et al., 2021; Wang et al., 2019). Massa et al. (2024) and Doyle et al. (2025) reported such a metric, dividing the search array into six pie-shaped “chunks” (each centered around a potential object location) and calculating the participant-level proportions of trials where the first saccade landed in the chunk containing the distractor (encoded as a binary trial-level outcome). We compared these participant-level proportions to the corresponding CNN accuracies to indirectly assess whether the information the network learns from is entirely described by the summary metric. If the CNN accuracy is not significantly greater than the proportion-level saccade landing rates, then the CNN may only be learning from information that can already be characterized using this metric. However, if the CNN outperforms this metric, it suggests that the network is learning from additional information not captured by this heuristic.

**Fig. 7:**
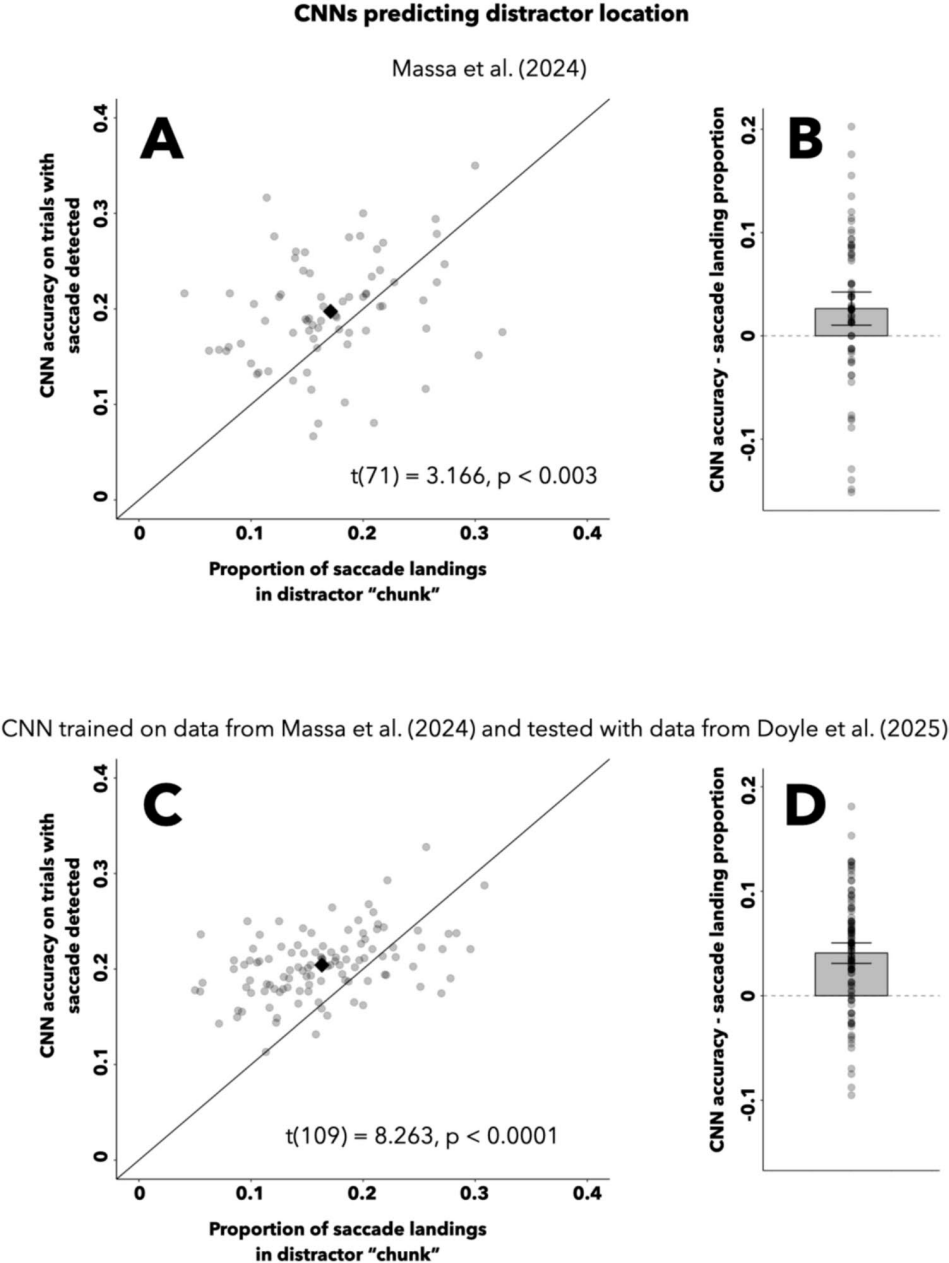
Comparison of CNN performance to a traditional oculomotor heuristic.** A**, **C** Participant-level averages of distractor-predicting CNN accuracy on trials where a saccade was detected, plotted against the corresponding proportions of saccade landings in the distractor-containing region, for the Massa et al. (2024) and Doyle et al. (2025) datasets (**A** and **C**, respectively). Diamond reflects mean across participants, and the unity line depicts points where the two values are equal. **B**, **D** Subject-level average differences between CNN accuracy and distractor-region-landing saccade proportions, plotted separately for the Massa et al. (2024) and Doyle et al. (2025) dataset (**B** and **D**, respectively). Bar plot depicts mean across participants, with a 95% confidence interval bootstrapped at the participant level

The classification accuracy of the CNN trained using data from Massa et al. (2024) was significantly higher than that of the heuristic using the rate of first saccades that land near the distractor. The CNN had an overall accuracy of 20.14% (mean across participants, 19.73%) on trials that contained eye data and a recorded saccade. Within this same subset, the first recorded saccade landed in the distractor-containing chunk on 17.01% of trials (mean proportion across participants, 17.10%). The participant-level CNN accuracies were significantly greater than the participant-level first saccade heuristic (Fig. 7A), *t*(71) = 3.166, *p* < 0.003. Moreover, the mean difference between participant-level CNN accuracy and first-saccade-landing heuristic (2.63%) had a 95% participant-level confidence interval [1.03–4.23%] that did not include 0 (Fig. 7B). Thus, the CNN trained to predict distractor location using eye data from Massa et al. (2024) outperformed the traditional heuristic using first saccade landings in distractor regions, suggesting that the CNN uses oculomotor information not captured by this metric when forming predictions.

When applied to the dataset from Doyle et al. (2025), the CNN trained using data from Massa et al. (2024) again predicted distractor location with a significantly higher accuracy than the heuristic, which uses the rate of first saccades landing in the distractor-containing “chunk”. The CNN’s overall accuracy was 20.64% (mean across participants, 20.41%) on trials from Doyle et al. (2025) that contained eye data and a recorded saccade, while the first recorded saccade landed in the distractor-containing chunk on 17.01% of these trials (mean across participants, 16.33%). Illustrated in Fig. 7C, the CNN participant-level accuracies were significantly greater than the corresponding first saccade landing rates, *t*(109) = 8.263, *p* < 0.0001. The mean difference between the participant-level CNN accuracy and participant-level saccade landing rates (4.09%) had a 95% bootstrapped, participant-level confidence interval [3.10–5.03%] that did not include 0 (Fig. 7D). These findings illustrate how the CNN again outperformed the traditional oculomotor metric in a higher-powered sample of trials, further indicating that the CNN does not solely use the landing of the first saccade when learning to form predictions about distractor location.

### Interim discussion II

In the previous section, we demonstrated how CNNs can learn from time-course eye position data to successfully predict the location of task-irrelevant search distractors, indicating the presence of oculomotor evidence for experience-driven attention within such data. While these results highlight the utility of our approach as a complement to traditional oculomotor analyses, they do not discern what “evidence” the CNNs actually learn from. The previous comparisons of CNN accuracy to traditional saccade-landing metrics provide some insight, that the CNNs are using more than just first saccade landings to form predictions, but not enough to identify the exact spatiotemporal patterns relevant for classification. To further uncover these patterns, we used SHAP analysis, a technique for visualizing “feature importance” that computes an additive metric quantifying each input feature’s specific contribution to a model’s output prediction (H. Chen et al., 2023; Lundberg & Lee, 2017). We applied SHAP analysis in the upcoming section (see “Methods—SHAP analysis” for more information) to interpret what the distractor-predicting CNNs actually learn from in the raw eye traces^4^. Specifically, we determined the eye position values most relevant to trial-level classifications and assessed their spatial and temporal distributions, comparing such distributions to relevant behavioral metrics and experimental design aspects.

### What information are the CNNs learning from to accurately form predictions?

#### Comparing feature importance to response times: a “sanity check”

**Fig. 8:**
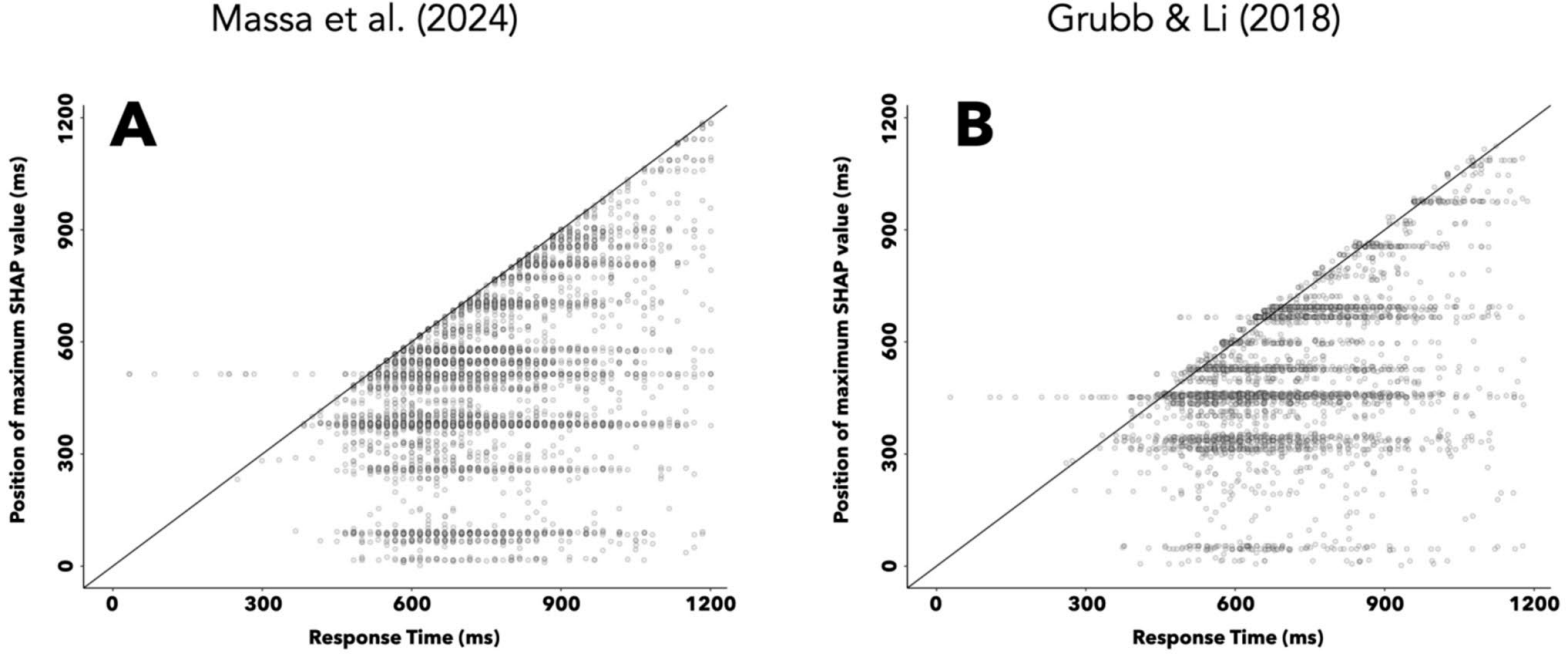
Temporal patterns in feature importance for distractor-predicting CNNs. **A**, **B** Unity plot depicting the position of the largest SHAP value in each trial against the observed RT, for a CNN predicting distractor location in the Massa et al. (2024) or Grubb and Li (2018) dataset (**A** and **B**, respectively). Each point represents an individual trial

As an initial foray into our SHAP analysis, we confirmed that distractor-predicting CNNs only used recorded eye position samples occurring before a response was made to form predictions. We extracted the position of the largest SHAP value (i.e., the most informative value within that trial’s time-course), and plotted this trial-level position metric against the corresponding behavioral response time (Fig. 8)^5^. For both CNNs (Massa et al., 2024, and Grubb & Li, 2018, in Fig. 8A and B, respectively), the vast majority of the points are on or below the unity line, indicating that the response time (RT) is greater than the position of the maximum SHAP value on those trials. This pattern of results confirms that the networks primarily relied on time-course data occurring before a response was made (i.e., recorded eye data on that trial) to generate predictions.

#### Characterizing spatiotemporal patterns in feature importance

**Fig. 9:**
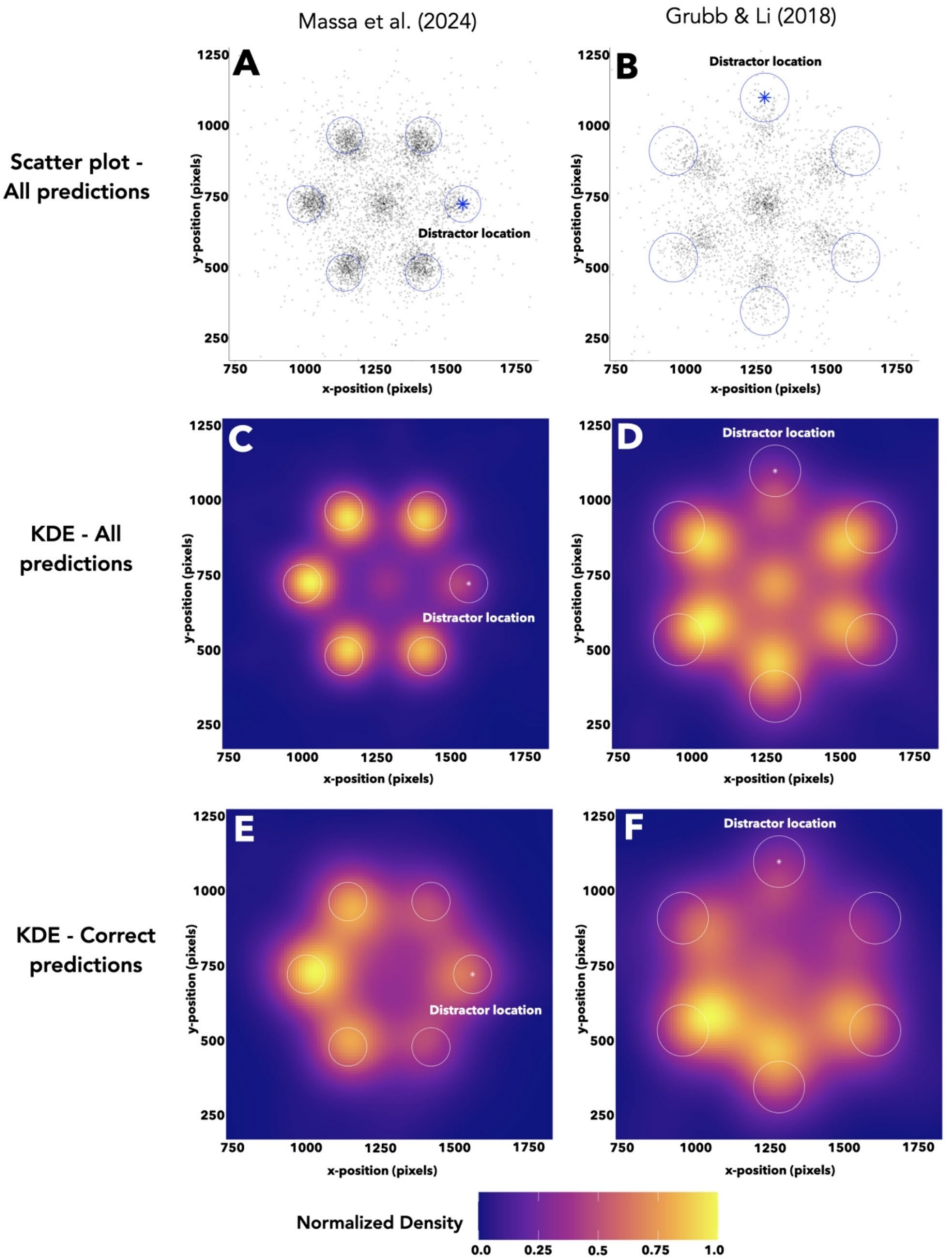
Spatial patterns in feature importance for distractor-predicting CNNs. **A**, **B** Plot of the gaze coordinates corresponding with the maximum SHAP value on each trial, after rotating all trials to align distractor locations, for CNNs predicting distractor location using data from Massa et al. (2024) and Grubb and Li (2018) (**A** and **B**, respectively). Circles denote possible object locations (radii drawn to scale, pixel coordinates from background study of Grubb and Li (2018) used in **B**); asterisks denote distractor location. **C**–**F** Heatmaps depicting results of Gaussian-weighted kernel density estimates for the spatial distributions of trial-level maximum SHAP values, for a CNN predicting distractor location using data from Massa et al. (2024) and Grubb and Li (2018) (former, **C** and **E**; latter, **D** and **F**). Circles denote possible object locations (radii drawn to scale, pixel coordinates from background study of Grubb and Li (2018) used in **D** and **F**); asterisks denote distractor location. Panels **C** and **D** plot the KDE for all trials, while panels **E** and **F** plot the KDE for trials with a correct CNN prediction

To assess spatial patterns in feature importance, we visualized the spatial distribution of the largest SHAP values on each trial after rotating all traces around the fixation point to align all distractor positions. On each trial of the validation set, we extracted the eye position sample that corresponded to the largest SHAP value (i.e.*,* the sample that was most informative for each trial-level classification). We then rotated the basis of each search array to align every distractor at the same position (e.g., Findlay, 1997; see Supplementary Methods for more information) and plotted the rotated positions of the trial-level maximum SHAP values (Fig. 9A and B). We then quantified regions of spatial importance via a Gaussian-weighted kernel density estimation (KDE) on these rotated trial-level maximum SHAP values (Fig. 9C–F; see Methods for more information).

While distractor-predicting CNNs are influenced by target location when forming predictions, they also learn from eye position values opposite of experience-driven distractors. Figure 9C depicts the KDE for the Massa et al. (2024) dataset (distractors on the right horizontal meridian). Graphically, it appears that the trial-level maximum SHAP values are clustered near every potential object location besides that of the distractor. Such a pattern on its own would suggest that the CNNs are relying on target location, which could be at any location besides that of the distractor, to form predictions by using the attentional signal towards targets to rule out that location and randomly guessing from the remaining five for an accuracy of ~20%. However, upon repeating this analysis for only trials in which the CNN correctly predicted distractor location (Fig. 9E), a different pattern emerges: The trial-level maximum SHAP values were most heavily concentrated at polar angles opposite the distractor. These results hold for the CNN predicting distractor location in the Grubb and Li (2018) dataset as well (Fig. 9D, all trials; Fig. 9F, correctly predicted trials; distractors on the upper vertical meridian). This pair of findings suggests that while the networks were influenced by the oculomotor signal of attention towards targets, they also detected and learned from a repulsive effect on gaze by experience-driven distractors.

In order to further characterize an oculomotor signal for experience-driven attention, we needed to disentangle trials where the CNN used this signal from trials where the network ruled out the target location and happened to guess correctly. One approach would be to repeat the above analysis using the subset of trials with a correct CNN prediction and a first saccade that does not land in the target location “chunk” (see “Results—Comparison of distractor-location CNN accuracy to traditional oculomotor metrics” for more information). The oculomotor signal for target location is more likely to be removed with this additional constraint, since a SHAP analysis on target-predicting CNNs (and general intuition) suggests that this signal arises from saccades towards targets just before a response (see Supplemental Results for full reporting). However, excluding trials with a first saccade towards the target from the previously used subset of correct-prediction trials risks insufficient power. Fortunately, our demonstration of transfer learning used the entire Doyle et al. (2025) dataset and required no additional network training, meaning that the learned weights used to generate predictions (which are involved in the computation of SHAP values) are shared across both networks. Thus, a SHAP analysis on the Doyle et al. (2025) dataset would serve as a higher-powered feature visualization for the CNN trained on the Massa et al. (2024) dataset. Isolating Doyle et al. (2025) trials with a correct CNN prediction and a non-target-directed first saccade produces a subset with sufficient power, allowing us to perform a SHAP analysis with a greater chance of isolating an oculomotor signal for experience-driven attention.

**Fig. 10:**
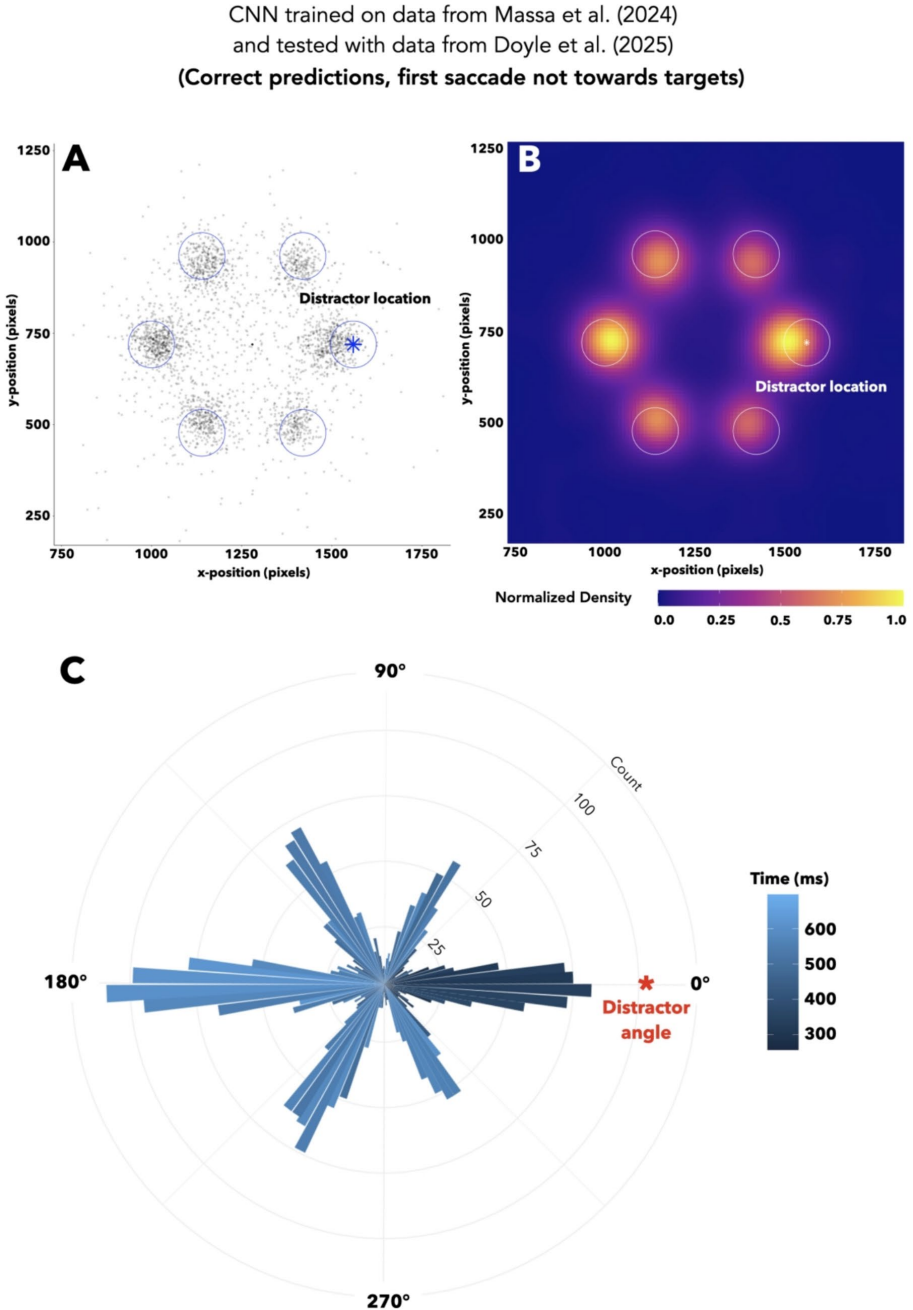
Spatiotemporal SHAP analysis in a higher-powered dataset. All figures reflect the resulting subset after excluding trials with a first saccade towards the target location and sub-setting for correct CNN predictions. **A** Plot of the gaze coordinates corresponding with the maximum SHAP value on each trial, after rotating all trials to align distractor locations, for the CNN trained on data from Massa et al. (2024) and predicting distractor location in the Doyle et al. (2025) dataset. Circles denote possible object locations (radii drawn to scale); asterisks denote distractor location. **B** Heat maps depicting results of Gaussian-weighted kernel density estimates for the spatial distributions of trial-level maximum SHAP values, for the CNN trained on data from Massa et al. (2024) and predicting distractor location in the Doyle et al. (2025) dataset. Circles denote possible object locations (radii drawn to scale); asterisks denote distractor location. **C** Histogram of maximum SHAP value position around polar angles, for the CNN trained on data from Massa et al. (2024) and predicting distractor location in the Doyle et al. (2025) dataset. Asterisk denotes the polar angle of the target, and the color of bars signifies the average time in the trial at which the maximum SHAP value occurred

A spatial SHAP analysis on the higher-powered Doyle et al. (2025) dataset found that the most informative eye position values were on the same and opposite polar angle as the distractor, with the former occurring earlier in time than the latter. We isolated trials in the Doyle et al. (2025) dataset with a correct CNN prediction and a first saccade not directed towards the target, and repeated our spatial SHAP analysis on this subset (Fig. 10A, B). As shown in the resulting KDE (Fig. 10B), the maximum SHAP values were most concentrated at the distractor location and the location opposite the distractor. Figure 10C depicts a histogram of the trial-level maximum SHAP values at different polar angles, with color reflecting the average trial-level time point of each sample within the bin. This figure illustrates how, on average, the maximum SHAP values near the distractor occurred earlier in time than those opposite the distractor. This pattern suggests that the CNNs learned from reflexive allocations of attention towards experience-driven distractors and later corrective shifts in gaze away from distractor location.

## Discussion

Traditional analyses of oculomotor data from visual search tasks rely on simplified metrics or heuristics to ensure tractability. Here, we develop a CNN-based analysis that can independently learn from the entirety of an oculomotor time-course, leveraging the complexity and richness contained within. We applied CNNs to three oculomotor datasets from previous visual search studies in our lab (Doyle et al., 2025; Grubb & Li, 2018; Massa et al., 2024). All datasets arose from the second phase of their respective studies, where participants searched for a shape-defined target among five distractors, one of which was rendered in a previously target-indicative color from the first phase. This distractor induced reflexive allocations of overt attention due to its “history as a sought target” (Anderson et al., 2021). We posited the following: If such distractors engender reflexive shifts in overt attention towards their location, evidence for these allocations should be present in the eye data, and a CNN should be able to learn from this evidence. Indeed, our CNN-based approach successfully classified task-irrelevant distractor location using data from Massa et al. (2024) and Grubb and Li (2018). We also demonstrated model generalizability, with a CNN trained on data from Massa et al. (2024) able to classify distractor location within the Doyle et al. (2025) dataset. These findings serve to validate our approach and highlight its utility as a complementary approach to traditional oculomotor analyses.

Our CNN-based approach identified oculomotor evidence for two different types of overt attention within the same dataset with minimal changes to the approach. In the two analyses applied to Massa et al. (2024) and Grubb and Li (2018), one CNN classified trials based on target location, and another classified trials based on distractor location. The only changes in procedure between these analyses were the sub-setting of distractor-present trials and the comparison of CNN classifications to the location of the distractor rather than the target. In other words, both CNNs were identical in architecture, and the CNN predicting target location was given all of the trials provided to the CNN predicting distractor location. Yet the former successfully classified the location of the target, and the latter successfully classified the location of the distractor. Just by changing the “correct answer” during learning, from the location of one search object to that of another, a CNN can use the same eye data to find evidence for an entirely different form of overt attention (e.g., endogenous vs. experience-driven). Importantly, the reflexive allocation of experience-driven attention was generated by a stimulus that was *entirely task-irrelevant and physically non-salient*, highlighting the value of our CNN-based approach’s ability to predict distractor location from raw eye traces.

The CNNs learned from aspects of the raw eye traces not captured by a traditional oculomotor metric, as demonstrated by comparing network performance to this metric and performing feature visualization. Measuring the frequency of first saccade landings at or near the distractor is a commonly used heuristic for quantifying the attentional impact of a task-irrelevant distractor during search (e.g., Doyle et al., 2025; Massa et al., 2024; Sauter et al., 2021; Wang et al., 2019). In multiple datasets (i.e*.*, Massa et al., 2024, and Doyle et al., 2025), a distractor-predicting CNN demonstrated a higher classification accuracy than the corresponding participant-level saccade landing rates, suggesting that the network learned from information not captured by this heuristic. Feature visualization further supported this conclusion, indicating that eye position samples opposite distractors and the trial-level timing of samples were also relevant for distractor classification. Thus, our complementary approach was able to identify aspects of an oculomotor signal for experience-driven attention not available in a traditional approach to interpreting eye data.

A successful demonstration of model generalizability supports the idea that the CNNs learn from oculomotor patterns indicative of reflexive overt attention, rather than overfitting to a specific dataset. By training a network on eye data from one participant set and successfully classifying distractor location using data from another set, we can eliminate the possibility that the networks are memorizing participant-specific noise to form predictions. Instead, since the relevant information for distractor location is shared between datasets, successful distractor classification using novel data substantiates the claim that the CNN is learning from an oculomotor signature of reflexive overt attention elicited by experience-driven distractors. When incorporating our approach, others could employ similar demonstrations of generalizability. Training a CNN on one dataset and testing on another (provided that the search arrays are identical) can confirm that the CNNs learn from converging evidence of overt attention that is shared across studies.

Our approach uses neural networks as an analysis tool rather than as a method of studying the visual system. Another use of CNNs within neuroscience links CNNs back to the biological visual system on which they were originally based (Krizhevsky et al., 2012; Lindsay, 2021). The aim with such a use is to create a single neural network that generalizes performance across multiple visual tasks and datasets, using this computational tool to further understand human vision (Marblestone et al., 2016; Richards et al., 2019). In an effort to achieve this goal, a CNN learns from as much data across as many different image types as possible, with its hyperparameters tuned to maximize its success (Gu et al., 2018). Our use of CNNs as a tool for oculomotor analysis differs from this indirect study of vision in aim and in practice. Rather than provide multiple datasets to one CNN and alter its structure to maximize performance across tasks, we created a new CNN for each task and observed the resulting performance of each network. Here, classification accuracy is not an indicator of the CNN’s quality as a representative model, but is instead a marker of whether oculomotor evidence for overt attentional allocations exists within time-course eye data. Thus, our complementary CNN-based approach to analyzing eye data is distinct from traditional models of the visual system both in application and interpretation.

With the nuances of the approach in mind, it is important to note that our developed analysis can only detect (and not exclude) the presence of oculomotor evidence for overt attentional allocations towards search objects. While a CNN can learn from evidence within eye data to classify search object location, a CNN that demonstrates chance-level accuracy in classification does not necessarily indicate a lack of such evidence. In such cases, this at-chance performance could also be attributed to the unchanged set of hyperparameters or to the internal architecture of the CNN not being optimal for classifying the applied dataset. However, there is no such ambiguity for CNNs demonstrating cross-validated above-chance classification accuracy; such a result is clearly indicative of oculomotor evidence for overt attentional shifts.

Our CNN-based approach does have some limiting factors in its application. One such limitation is the need for pre-defined search target locations. There do exist paradigms where the location of the search target can appear nearly anywhere on the screen (Wolfe, 2020). If one were to apply our CNN-based approach to such a protocol, the number of classes would be equivalent to the pixel area of the screen on which the task was performed! Such an application is clearly impractical with our current CNN architecture. However, other CNN architectures such as U-nets (Ronneberger et al., 2015) can efficiently produce predictions of images and thus might plausibly be adapted to such a task. Additionally, our approach relies on participants making overt allocations of attention during a search. Participants could theoretically employ covert attention strategies to perform the search task, eliminating any oculomotor evidence for a CNN to learn from and producing chance-level network classification accuracy. While we can eliminate the possibility of an *entirely* covert attention strategy used by participants in the current applications (as all networks demonstrated above-chance accuracy), the need for overt attentional shifts during search is a limiting factor of the approach.

Future applications of this CNN-based approach to oculomotor analysis can focus on other variables within eye data, such as pupil size. Moment-to-moment changes in pupil area have been linked to a variety of cognitive processes, such as arousal, preparation of eye movements, and subjective interpretation of visual stimuli (Mathot, 2018; Sirois & Brisson, 2014). The speed at which pupil area changes can also contain important information about changes in ongoing attentional processes (for a review, see Mathot, 2018). Many commercial eye trackers have the capability to record changes in pupil area in the same time-course structure as eye position. Future work could apply a CNN-based analysis to pupil area data gathered from protocols formally investigating cognitive processes through pupillometry.

For the scientist studying visual search, a CNN-based approach is a valuable option as a complementary method of analyzing eye data. CNNs can classify the location of search objects from time-course eye position data, with the resulting accuracy in classification acting as a dependent variable to which a variety of analyses can be applied. These networks can evidence multiple forms of attention, both task-relevant and task-irrelevant, within the same eye data and with minimal changes to the approach. Furthermore, training on one set of eye data is generalizable across oculomotor datasets from different studies, indicative of a shared oculomotor signature for reflexive overt attention. Through feature visualization, our approach is able to characterize the spatiotemporal patterns that make up this signature, which includes aspects of the gaze data ignored by traditional oculomotor heuristics. Finally, our approach preserves the spatiotemporal complexity of time-course recordings and minimizes the need for data exclusion and summary metrics during analysis. The use of convolutional neural networks that classify target location or other experimental stimuli would thus serve well the scientist searching for a complementary analysis of visual search oculomotor data.

## Supplementary Information

Below is the link to the electronic supplementary material.

Supplementary file1 (DOCX 8.61 MB)

## Data Availability

All data can be found at 10.5281/zenodo.19489021.

## Footnotes

1 Here, we include participants who received accuracy-based feedback during the training phase in both the “Background Study” and the “Registered Report.” The 24 participants in the background study who received reward-based feedback are not included.

2 Since the stimulus sizes and search array were coded using degrees of visual angle in Grubb and Li (2018), this change in viewing distance has no effect on how the search arrays were perceived by the participant. However, given that the *x*- and *y*-position values of eye position were coded in pixels, this difference does affect comparisons between eye position and search object location (see “Results—Characterizing spatiotemporal patterns in feature importance”).

3 The mean classification accuracy across participants is equal to the overall classification accuracy because all participants contributed the same number of trials to the validation dataset.

4 We also applied a SHAP analysis to the target-predicting CNNs, visualizing spatiotemporal patterns in feature importance for predicting search target location. In short, the most informative eye position values for target classification were clustered near the target location and occurred around the time a response was made. See Supplementary Results for full reporting.

5 Some trials with a negative RT occurred in Grubb and Li (2018) due to the method of encoding response time in this study. When participants made a response within ~16 ms (a singular frame of the testing room monitor), temporal misalignments between the signal indicating the opening of the response window and the response signal caused the RT to be recorded as negative. Such RTs occurred on only 13 total trials, making up ~0.1% of the Grubb and Li (2018) dataset (after nonresponses were excluded), and (along with nonresponses) were excluded from analysis.

## Code availability

The code for the CNN-based analyses and the statistical interpretation of model results can be found at https://github.com/nicholasdcrotty/CDVMBG_BRM_CNNOculomotorAnalysis.

## Declarations

### Open practices statement

The code for the CNN-based analyses and the statistical interpretation of model results can be found at https://github.com/nicholasdcrotty/CDVMBG_BRM_CNNOculomotorAnalysis. All data can be found at 10.5281/zenodo.19489021. None of the reported analyses were preregistered.
