# Supplementary material for: Deep learning and eye tracking: Convolutional neural networks provide converging evidence for experience-driven attention within visual search
Source: Behav Res Methods. 2026 Jun 4;58(7):187. doi: 10.3758/s13428-026-03057-2 (PMC13236786; doi:10.3758/s13428-026-03057-2)
Supplement: Supplementary file 1 — Supplementary file1 (DOCX 8.61 MB) [file 13428_2026_3057_MOESM1_ESM.docx]

SUPPLEMENTARY MATERIALS

# Supplementary Methods

## Section S1. Constructing a rotation matrix to align distractors

To assess the spatial distribution of SHAP values, we aligned the search array from each trial so that every distractor was positioned at the same location. To do so, we rotated the space described by each trial’s search array to align all distractors at one of the six potential locations (the “basis location”, which was chosen to be the location on the right horizontal meridian for Massa *et al.* (2024) and the upper vertical meridian for Grubb & Li (2018)). Such a rotation can be described by matrix $M$,

$M=\left[ \begin{matrix} cos(\theta) & sin(\theta) \\ -sin(\theta) & cos(\theta) \end{matrix} \right]$

where $\theta$ is the difference in polar angle between the basis location and the current trial’s distractor location. We can represent the trial-level time-course eye data as matrix $E$,

$$E=\left[ \begin{matrix} x_{1} & y_{1} \\ x_{2} & y_{2} \\ \vdots& \vdots\\ x_{r} & y_{r} \end{matrix} \right]$$

containing the *x*- and *y*-position of participants’ gaze $(x_{n},y_{n})$ from the onset of the search array until the time at which a response was made $(r)$. Since each rotation needs to be applied relative to central fixation, we can apply this rotation to the matrix of recorded eye traces through the following equation:

$$E^{*}=M(E-c)+c$$

where $E^{*}$ contains the rotated eye traces and $c$ is a 2-dimensional vector containing the pixel (*x*,*y*) coordinates of the central fixation point.

# Supplementary Results

## Section S1. Example predefined heuristic to predict target location

A “distance minimization” algorithm could also identify the location of the search target using the raw eye position data from Massa et al. (2024). For every eye position sample in each trial’s time-course, we calculated the distance (in pixels) of participants’ gaze from each of the six pixel locations where a target could potentially be displayed. Missing samples are regarded as NA (as opposed to being replaced with the origin as in the CNN-based analyses), and so the corresponding distance values for missing samples returned as NA. We then averaged these distances across samples (ignoring NA values), producing the trial-level average distance of participants’ gaze from the six potential locations. The potential location with the smallest observed distance is then returned and compared to the actual target location on that trial. Correct identifications occur when the location with the minimum distance matches the target location, while incorrect identifications occur when these two values do not align. On trials with no eye position data, a random selection from the six potential locations was returned as the identification (with the seed in RStudio set to 1823). Applying this predefined heuristic to the entire dataset from Massa et al. (2024) resulted in an overall accuracy of 60.39%. At the participant level, the mean accuracy across participants (60.39%) has a bootstrapped confidence interval ([55.23%–65.54%]) that is well above chance (16.67%), and a one-sample t-test indicates that the participant-level mean accuracies are significantly above chance (t(71) =16.17, p < 0.0001). This analysis exemplifies how pre-defined heuristics can still identify search target location with a relatively simple approach.

## Section S2. SHAP analysis of target-predicting CNNs

A spatial SHAP analysis indicated the target-predicting CNNs learned from direct fixations on or near the search target to make predictions. As done with distractor-predicting CNNs, we rotated the trial-level eye traces from all CNN predictions (provided a response was made) on the same search array to align the target at the same position in the array (e.g., Findlay, 1997b), and plotted the trial-level maximum SHAP values (*i.e.*, the eye position sample that was most informative for the classification of that trial) onto the search arrays. **Figure S1A** depicts such a plot for the Massa *et al.* (2024) and dataset, with all targets positioned at the location on the right horizontal meridian. **Figure S1B** shows the same plot for the Grubb & Li (2018) datasets, with all targets on the upper vertical meridian. Even from the scatter plots alone, it is apparent that the most informative eye position values on each trial are clustered around the target. A Gaussian-weighted kernel density estimation (KDE) also demonstrates this pattern in the two datasets, both for all trials (**Figures S1C-S1D**), and correct CNN predictions (**Figures S1E-S1F**).

Both CNNs only used recorded eye-position samples occurring before a response was made to form predictions. As a “sanity check”, we extracted the position of the largest SHAP value (i.e., the most informative value within that trial’s time-course), and plotted this trial-level position metric against the corresponding behavioral response time (**Figures S2A, S2C**), excluding nonresponses and trials with negative RTs^^[[1]](#footnote-2)^^. For both CNNs predicting target location, (Massa et al. (2024): **Figure S2A**, Grubb & Li (2018): **Figure S2C**) the vast majority of the points are on or below the unity line, indicating that the response time (RT) is greater than the position of the maximum SHAP value on those trials. This pattern of results confirm that the networks only used time-course data occurring before a response was made (*i.e.*, recorded eye data on that trial) to generate predictions.

The eye position values that were most informative for target-location classification occurred around the time a response was made. To visualize the pattern of SHAP values as each time-course approaches the observed RT, we time-locked each trial’s set of SHAP values to align every RT at the same position in the time-course. This realignment meant that all trials besides that with the longest RT would need to be padded at the beginning with NAs. Due to slight temporal misalignments between the frame rate of the testing room screen and the signal indicating the response window’s end, trials with an RT longer than the 1200ms window were regarded as 1200ms. We then averaged these realigned SHAP values across trials to obtain a “representative trial” for each study’s validation dataset, as depicted in **Figures S2B** and **9D** (Massa *et al.*, (2024): **Figure S2B**, Grubb & Li (2018): **Figure S2D**). Ignoring the noise at early timepoints (caused by little data at these timepoints due to differing trial lengths), we can see that the largest SHAP values in each plot occur at the end of this aligned time-course (*i.e.*, just prior to the RT). In other words, the eye position values just before a response was made are the most relevant and informative for classification. Such a relationship is relatively intuitive, as participants will most likely be fixating on the target while they perform an orientation judgement and input a response.


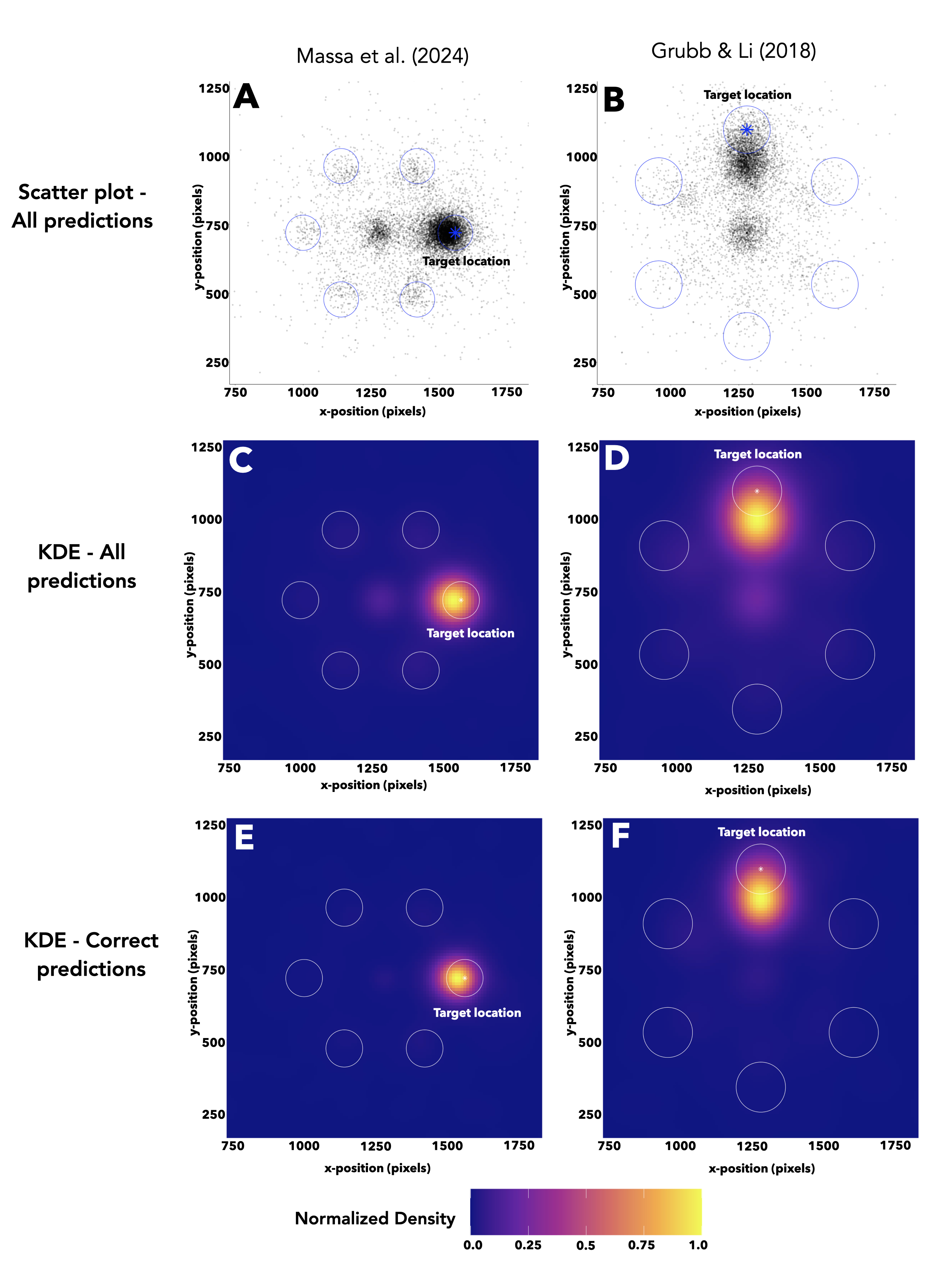


**Figure S1. *Spatial patterns in feature importance for target-predicting CNNs.*** A-B) Plot of the gaze coordinates corresponding with the maximum SHAP value on each trial, after rotating all trials to align target locations, for CNNs predicting target location using data from Massa *et al.* (2024) and Grubb & Li (2018) (A and B, respectively). Circles denote possible object locations (radii drawn to scale, pixel coordinates from background study of Grubb & Li (2018) used in B), asterisks denote target location. C-F). Heatmaps depicting results of Gaussian-weighted kernel density estimates for the spatial distributions of trial-level maximum SHAP values, for a CNN predicting target location using data from Massa *et al.* (2024) and Grubb & Li (2018) (former, C and E; latter, D and F). Circles denote possible object locations (radii drawn to scale, pixel coordinates from background study of Grubb & Li (2018) used in D and F), asterisks denote target location. Figures C and D plot the KDE for all trials, while Figures E and F plot the KDE for trials with a correct CNN prediction.


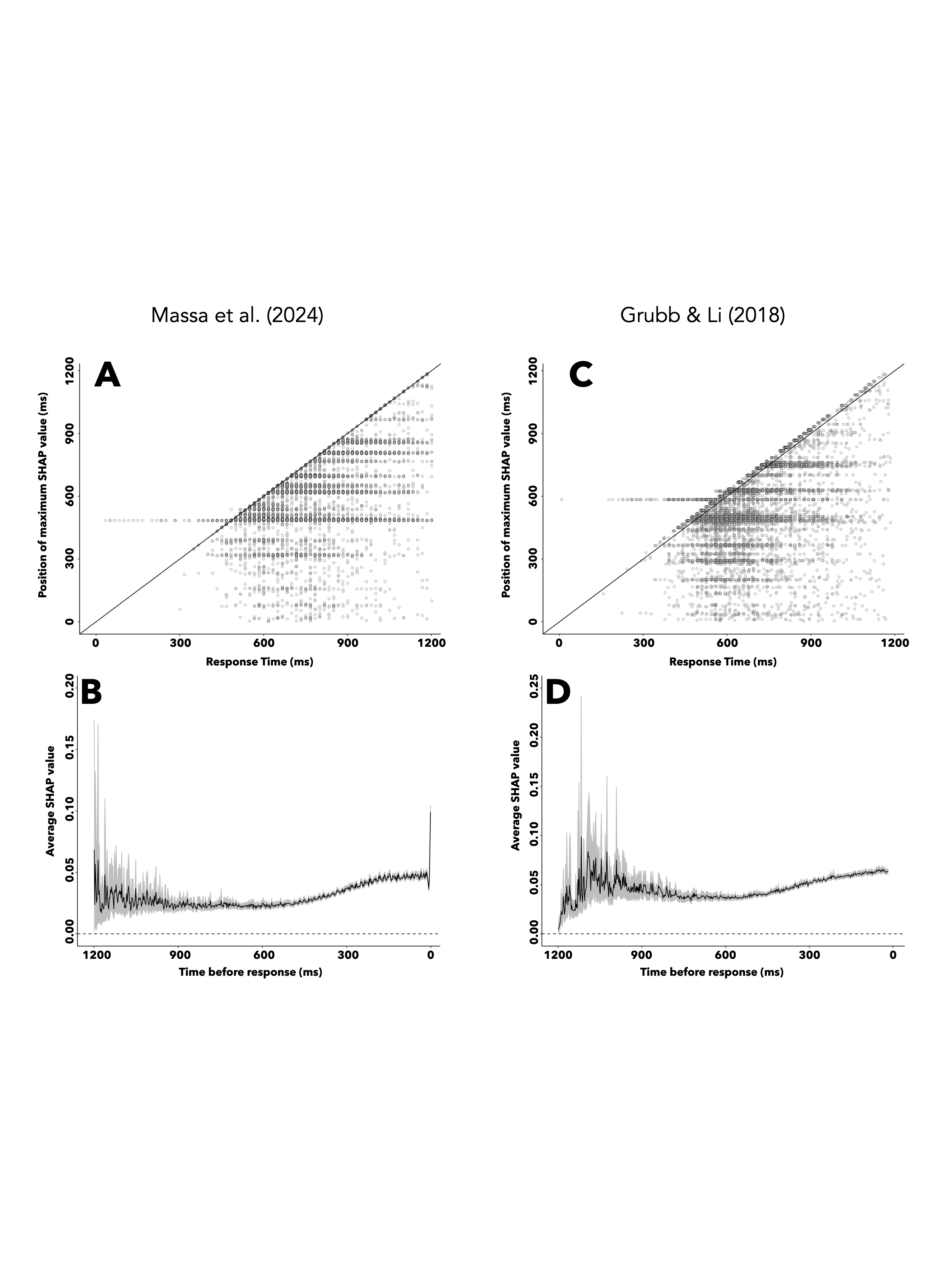


**Figure S2.** ***Temporal patterns in feature importance for target-predicting CNNs.*** A,C) Unity plot depicting the position of the largest SHAP value in each trial against the observed RT, for a CNN predicting target location in the Massa *et al.* (2024) or Grubb & Li (2018) dataset (A and C, respectively). Each point represents an individual trial. B,D) Averaged re-aligned time-courses (relative to the observed RT) of SHAP values for a CNN predicting target location in the Massa *et al.* (2024) or Grubb & Li (2018) dataset (A and C, respectively). Dashed line represents SHAP values of 0 (*i.e.*, samples that are entirely uninformative for classification). Shaded region represents a bootstrapped trial-level confidence interval for each sample.

# Open Practices Statement

The code for the CNN-based analyses and the statistical interpretation of model results can be found at <https://github.com/nicholasdcrotty/CDVMBG_BRM_CNNOculomotorAnalysis>. All data can be found at <https://doi.org/10.5281/zenodo.19489021>. None of the reported analyses were preregistered.

# Declarations

## Funding

Supported by NSF-2141860 CAREER Award to MAG, NIH grant R01EY033628 to NCB, and NSF GRFP to NC.

## Conflicts of Interest/Competing Interests

Nothing to report.

## Ethics approval

The data analyzed in this manuscript arose from three separate projects that all received ethical approval by the Trinity College Institutional Review Board.

## Consent to participate

Informed consent was obtained for each participant.

## Consent for publication

Not applicable.

## Availability of data and materials

All data can be found at <https://doi.org/10.5281/zenodo.19489021>.

## Code availability

The code for the CNN-based analyses and the statistical interpretation of model results can be found at <https://github.com/nicholasdcrotty/CDVMBG_BRM_CNNOculomotorAnalysis>.

# References

Anderson, B. A., Kim, H., Kim, A. J., Liao, M. R., Mrkonja, L., Clement, A., & Gregoire, L. (2021). The past, present, and future of selection history. *Neurosci Biobehav Rev, 130*, 326-350. doi:10.1016/j.neubiorev.2021.09.004

Anderson, B. A., & Yantis, S. (2012). Value-driven attentional and oculomotor capture during goal-directed, unconstrained viewing. *Atten Percept Psychophys, 74*(8), 1644-1653. doi:10.3758/s13414-012-0348-2

Ansel, J., Yang, E., He, H., Gimelshein, N., Jain, A., Voznesensky, M., . . . Chintala, S. (2024). *PyTorch 2: Faster Machine Learning Through Dynamic Python Bytecode Transformation and Graph Compilation*. Paper presented at the Proceedings of the 29th ACM International Conference on Architectural Support for Programming Languages and Operating Systems, Volume 2, La Jolla, CA, USA. https://doi.org/10.1145/3620665.3640366

Becker, S. I., Ansorge, U., & Horstmann, G. (2009). Can intertrial priming account for the similarity effect in visual search? *Vision Research, 49*(14), 1738-1756.

Benson, N. C., Song, B., Chen, S., Miyata, T., Takemura, H., & Winawer, J. (2025). Machine Learning Matches Human Performance at Segmenting the Human Visual Cortex. *bioRxiv*, 2025.2005.2016.654503. doi:10.1101/2025.05.16.654503

Bisong, E. (2019). Regularization for Deep Learning. In *Building Machine Learning and Deep Learning Models on Google Cloud Platform: A Comprehensive Guide for Beginners* (pp. 415-421). Berkeley, CA: Apress.

Chen, H., Covert, I. C., Lundberg, S. M., & Lee, S.-I. (2023). Algorithms to estimate Shapley value feature attributions. *Nature Machine Intelligence, 5*(6), 590-601. doi:10.1038/s42256-023-00657-x

Chen, Y.-C. (2017). A tutorial on kernel density estimation and recent advances. *Biostatistics & Epidemiology, 1*(1), 161-187. doi:10.1080/24709360.2017.1396742

Doyle, A., Volkova, K., Crotty, N., Massa, N., & Grubb, M. A. (2025). Information-driven attentional capture. *Atten Percept Psychophys, 87*(3), 721-727. doi:10.3758/s13414-024-03008-z

Duane, S., Kennedy, A. D., Pendleton, B. J., & Roweth, D. (1987). Hybrid Monte Carlo. *Physics Letters B, 195*(2), 216-222. doi:https://doi.org/10.1016/0370-2693(87)91197-X

Findlay, J. M. (1997). Saccade Target Selection During Visual Search. *Vision Research, 37*(5), 617-631. doi:https://doi.org/10.1016/S0042-6989(96)00218-0

Fukushima, K. (1980). Neocognitron: a self organizing neural network model for a mechanism of pattern recognition unaffected by shift in position. *Biol Cybern, 36*(4), 193-202. doi:10.1007/BF00344251

Gaspelin, N., Ruthruff, E., & Lien, M.-C. (2016). The problem of latent attentional capture: Easy visual search conceals capture by task-irrelevant abrupt onsets. *Journal of Experimental Psychology: Human Perception and Performance, 42*(8), 1104.

Godwin, H. J., Hout, M. C., Alexdóttir, K. J., Walenchok, S. C., & Barnhart, A. S. (2021). Avoiding potential pitfalls in visual search and eye-movement experiments: A tutorial review. *Attention, Perception, & Psychophysics, 83*(7), 2753-2783.

Grubb, M. A., & Li, Y. (2018). Assessing the role of accuracy-based feedback in value-driven attentional capture. *Atten Percept Psychophys, 80*(4), 822-828. doi:10.3758/s13414-018-1494-y

Gu, J., Wang, Z., Kuen, J., Ma, L., Shahroudy, A., Shuai, B., . . . Cai, J. (2018). Recent advances in convolutional neural networks. *Pattern recognition, 77*, 354-377.

Hochreiter, S. (1991). Untersuchungen zu dynamischen neuronalen Netzen. *Diploma, Technische Universität München, 91*(1), 31.

Hollingworth, A., & Bahle, B. (2020). Eye tracking in visual search experiments. *Spatial learning and attention guidance*, 23-35.

Joseph, V. R. (2022). Optimal ratio for data splitting. *Statistical Analysis and Data Mining: The ASA Data Science Journal, 15*(4), 531-538.

Kim, B., Reif, E., Wattenberg, M., Bengio, S., & Mozer, M. C. (2021). Neural Networks Trained on Natural Scenes Exhibit Gestalt Closure. *Computational Brain & Behavior, 4*(3), 251-263. doi:10.1007/s42113-021-00100-7

Kingma, D., & Ba, J. (2015). *Adam: A Method for Stochastic Optimization*. Paper presented at the International Conference for Learning Representations, San Diego, CA.

Krizhevsky, A., Sutskever, I., & Hinton, G. E. (2012). Imagenet classification with deep convolutional neural networks. *Advances in neural information processing systems, 25*.

Kruper, J., Richie-Halford, A., Benson, N. C., Caffarra, S., Owen, J., Wu, Y., . . . Consortium, U. K. B. E. V. (2024). Convolutional neural network-based classification of glaucoma using optic radiation tissue properties. *Communications Medicine, 4*(1), 72. doi:10.1038/s43856-024-00496-w

Kruper, J., Richie-Halford, A., Benson, N. C., Caffarra, S., Owen, J., Wu, Y., . . . U.K. Biobank Eye and Vision Consortium. (2024). Convolutional neural network-based classification of glaucoma using optic radiation tissue properties. *Commun Med (Lond), 4*(1), 72. doi:10.1038/s43856-024-00496-w

Kullback, S., & Leibler, R. A. (1951). On information and sufficiency. *The annals of mathematical statistics, 22*(1), 79-86.

LeCun, Y., Boser, B., Denker, J. S., Henderson, D., Howard, R. E., Hubbard, W., & Jackel, L. D. (1989). Backpropagation Applied to Handwritten Zip Code Recognition. *Neural Computation, 1*(4), 541-551. doi:10.1162/neco.1989.1.4.541

Lindsay, G. W. (2021). Convolutional neural networks as a model of the visual system: Past, present, and future. *Journal of cognitive neuroscience, 33*(10), 2017-2031.

Lundberg, S. M., & Lee, S.-I. (2017). A unified approach to interpreting model predictions. *Advances in neural information processing systems, 30*.

Mao, A., Mohri, M., & Zhong, Y. (2023). *Cross-entropy loss functions: Theoretical analysis and applications.* Paper presented at the International conference on Machine learning.

Marblestone, A. H., Wayne, G., & Kording, K. P. (2016). Toward an Integration of Deep Learning and Neuroscience. *Front Comput Neurosci, 10*, 94. doi:10.3389/fncom.2016.00094

Massa, N. B., Crotty, N., Levy, I., & Grubb, M. A. (2024). Manipulating the reliability of target-color information modulates value-driven attentional capture. *Attention, Perception, & Psychophysics, 86*(4), 1108-1119.

Mathot, S. (2018). Pupillometry: Psychology, Physiology, and Function. *J Cogn, 1*(1), 16. doi:10.5334/joc.18

McCulloch, W. S., & Pitts, W. (1943). A logical calculus of the ideas immanent in nervous activity. *The bulletin of mathematical biophysics, 5*(4), 115-133. doi:10.1007/BF02478259

McElreath, R. (2020). *Statistical Rethinking: A Bayesian Course with Examples in R and STAN* (2nd ed.). CRC Press.

Pierce, J., & MacAskill, M. (2018). *Building Experiments in PsychoPy*: SAGE.

Prechelt, L. (1998). Early Stopping - But When? In G. B. Orr & K.-R. Müller (Eds.), *Neural Networks: Tricks of the Trade* (pp. 55-69). Berlin, Heidelberg: Springer Berlin Heidelberg.

Rice, L., Wong, E., & Kolter, Z. (2020). *Overfitting in adversarially robust deep learning.* Paper presented at the International conference on machine learning.

Richards, B. A., Lillicrap, T. P., Beaudoin, P., Bengio, Y., Bogacz, R., Christensen, A., . . . Kording, K. P. (2019). A deep learning framework for neuroscience. *Nat Neurosci, 22*(11), 1761-1770. doi:10.1038/s41593-019-0520-2

Ronneberger, O., Fischer, P., & Brox, T. (2015). *U-net: Convolutional networks for biomedical image segmentation.* Paper presented at the Medical image computing and computer-assisted intervention–MICCAI 2015: 18th international conference, Munich, Germany, October 5-9, 2015, proceedings, part III 18.

Rosenblatt, F. (1958). The perceptron: a probabilistic model for information storage and organization in the brain. *Psychol Rev, 65*(6), 386-408. doi:10.1037/h0042519

Rumelhart, D. E., Hinton, G. E., & Williams, R. J. (1986). Learning representations by back-propagating errors. *Nature, 323*(6088), 533-536. doi:10.1038/323533a0

Sauter, M., Hanning, N. M., Liesefeld, H. R., & Müller, H. J. (2021). Post-capture processes contribute to statistical learning of distractor locations in visual search. *Cortex, 135*, 108-126. doi:https://doi.org/10.1016/j.cortex.2020.11.016

Shrikumar, A., Greenside, P., & Kundaje, A. (2017). *Learning important features through propagating activation differences.* Paper presented at the International conference on machine learning.

Silverman, B. W. (1986). *Density estimation for statistics and data analysis*: Routledge.

Sirois, S., & Brisson, J. (2014). Pupillometry. *Wiley Interdiscip Rev Cogn Sci, 5*(6), 679-692. doi:10.1002/wcs.1323

Sunde, B. M. (2024). early-stopping-pytorch: A PyTorch utility package for Early Stopping. Retrieved from https://github.com/Bjarten/early-stopping-pytorch

Wang, B., Samara, I., & Theeuwes, J. (2019). Statistical regularities bias overt attention. *Attention, Perception, & Psychophysics, 81*(6), 1813-1821. doi:10.3758/s13414-019-01708-5

Wolfe, J. M. (2020). Visual Search: How Do We Find What We Are Looking For? *Annu Rev Vis Sci, 6*, 539-562. doi:10.1146/annurev-vision-091718-015048

Wu, Y.-Y., Hu, Y.-S., Wang, J., Zang, Y.-F., & Zhang, Y. (2022). Toward precise localization of abnormal brain activity: 1D CNN on single voxel fMRI time-series. *Frontiers in Computational Neuroscience, 16*, 822237.

1. Trials with a negative RT only occurred in the Grubb & Li (2018) due to the method of encoding response time in this study. When participants made a response within ~16ms (a singular frame of the testing room monitor), temporal misalignments between the signal indicating the opening of the response window and the response signal caused the RT to be recorded as negative. Such RTs occurred on only 13 total trials, making up ~0.1% of the Grubb & Li (2018) dataset (after nonresponses were excluded). [↑](#footnote-ref-2)
